# The role and mechanism of TXNDC5 in disease progression

**DOI:** 10.3389/fimmu.2024.1354952

**Published:** 2024-04-02

**Authors:** Mingxia Jiao, Yeyong Zhang, Xie Song, Bing Xu

**Affiliations:** ^1^ Department of Urology, The First Affiliated Hospital of Shandong First Medical University & Shandong Province Qianfoshan Hospital, Shandong Medicine and Health Key Laboratory of Organ Transplantation and Nephrosis, Shandong Institute of Nephrology, Jinan, Shandong, China; ^2^ Shandong Provincial Key Laboratory for Rheumatic Disease and Translational Medicine, The First Affiliated Hospital of Shandong First Medical University & Shandong Provincial Qianfoshan Hospital, Jinan, Shandong, China; ^3^ Department of Orthopedic Surgery, The First Affiliated Hospital of Shandong First Medical University & Shandong Provincial Qianfoshan Hospital, Shandong Key Laboratory of Rheumatic Disease and Translational Medicine, Jinan, Shandong, China; ^4^ Department of Hepatobiliary Surgery, Shandong Provincial Hospital affiliated to Shandong First Medical University, Jinan, Shandong, China

**Keywords:** TXNDC5, inflammatory, rheumatoid arthritis, organ fibrosis, tumor

## Abstract

Thioredoxin domain containing protein-5 (TXNDC5), also known as endothelial protein-disulfide isomerase (Endo-PDI), is confined to the endoplasmic reticulum through the structural endoplasmic reticulum retention signal (KDEL), is a member of the PDI protein family and is highly expressed in the hypoxic state. TXNDC5 can regulate the rate of disulfide bond formation, isomerization and degradation of target proteins through its function as a protein disulfide isomerase (PDI), thereby altering protein conformation, activity and improving protein stability. Several studies have shown that there is a significant correlation between TXNDC5 gene polymorphisms and genetic susceptibility to inflammatory diseases such as rheumatoid, fibrosis and tumors. In this paper, we detail the expression characteristics of TXNDC5 in a variety of diseases, summarize the mechanisms by which TXNDC5 promotes malignant disease progression, and summarize potential therapeutic strategies to target TXNDC5 for disease treatment.

## 1 Introduction

The thioredoxin domain containing protein-5 (TXNDC5), also known as ERP46 (1), HCC-2, STRF8, PDIA15, UNQ364, endo PDI, is a member of the PDI family that is located on chromosome 6p24.3. Full-length cDNA analysis indicated that TXNDC5 is a 48 kDa protein measuring 845.2 kbp. It can encode five splice variants, of which TXNDC5-001 and TXNDC5-003 can be translated into proteins (2). TXNDC5 is widely distributed across tissues, mainly in the brain, spleen, lung, liver, kidney, pancreas, testis and others. TXNDC5 is highly expressed in endothelial cells and in the endothelium of tumors and atherosclerotic plaques, and TXNDC5 is upregulated in hypoxic conditions (1, 3–5). It is also upregulated in autoimmune diseases such as rheumatoid arthritis and highly expressed in various organ fibrosis diseases. Previous studies have shown that TXNDC5, like other PDI family members, regulates disulfide bond formation and rearrangement through the CxxC motif and assists in the proper folding of oxidized residue disulfide bonds (5–7). TXNDC5 functions as a stress survival factor and is required for endothelial cell survival under hypoxic conditions (3). TXNDC5 can mediate tumor necrosis factor-α (TNF-α) induced angiogenesis (8). TXNDC5 can bind to lipocalin receptor 1 as a cellular adapter and participate in cellular metabolism and inflammatory responses by activating downstream inflammatory factors (9). TXNDC5 can bind to alpha -mannosidase–like protein 3 (EDEM3) and trigger the mannose trimming activity of ER degradation to correct misfolded proteins (10). In addition, TXNDC5 also plays a molecular chaperone role and act synergistically with HSC70 to promote inflammation through NF-κB signal transduction (11). The association between TXNDC5 and disease susceptibility has been extensively reported, and multiple SNP across the TXNDC5 locus are closely associated with disease development (**Table 1**). In this review, we focus on the role of TXNDC5 in various diseases and discuss possible TXNDC5 applications for “TXNDC5-related diseases” particularly cancer, rheumatoid and fibrotic diseases.

**Table 1:** TXNDC5 SNPs and disease susceptibility.

Disease susceptibility	SNPs	Gene: Consequence	Ref
Cervical carcinoma	rs408014, rs7771314	BLOC1S5-TXNDC5: Intron Variant, TXNDC5: Intron Variant	(12, 13)
Liver cancer	rs13210097	BLOC1S5-TXNDC5: Intron Variant, PIP5K1P1: Non Coding Transcript Variant	(13)
	rs11754300	BLOC1S5-TXNDC5: Intron Variant, PIP5K1P1: Non Coding Transcript Variant	
	rs9392182	BLOC1S5-TXNDC5: Intron Variant	
	rs2815128	BLOC1S5: Intron Variant, BLOC1S5-TXNDC5: Intron Variant, EEF1E1-BLOC1S5: Intron Variant	
Oesophageal cancer	rs1632346, rs9505309	BLOC1S5-TXNDC5: Intron Variant	(13)
	rs2815128, rs2815142	BLOC1S5: Intron Variant, BLOC1S5-TXNDC5: Intron Varian, EEF1E1-BLOC1S5: Intron Variant	
Rheumatoid arthritis(RA)	rs1225936, rs1225938, rs2743992, rs372578, rs408014	BLOC1S5-TXNDC5: Intron Variant, TXNDC5: Intron Variant	(14)
	rs2743992	BLOC1S5: Intron Variant, BLOC1S5-TXNDC5: Intron Varian, EEF1E1-BLOC1S5: Intron Variant	
	rs41302895	BLOC1S5-TXNDC5: Non Coding Transcript Variant, BMP6: 3 Prime UTR Variant, TXNDC5: 3 Prime UTR Variant	
	rs9392189	BLOC1S5: Intron Variant, BLOC1S5-TXNDC5: Intron Variant, EEF1E1-BLOC1S5: Intron Variant	
	rs9505298	BLOC1S5-TXNDC5: 500B Downstream Variant, BMP6: 3 Prime UTR Variant, TXNDC5: 500B Downstream Variant	
Ankylosing spondylitis (AS)	rs1225937, rs1225938, rs372578, rs89715, rs378963, rs1225944 rs1225947, rs1238994, rs69086, rs408014, rs368074, rs1225954 rs1225955, rs13209404	BLOC1S5-TXNDC5: Intron Variant, TXNDC5: Intron Variant	(14)
	rs1044104	BLOC1S5-TXNDC5: 500B Downstream Variant, BMP6: 3 Prime UTR Variant, TXNDC5: 500B Downstream Variant	
	rs3812162	BLOC1S5-TXNDC5: Intron Variant, TXNDC5: 2KB Upstream Variant	
Non-segmental vitiligo (NSV)	rs1043784	BLOC1S5-TXNDC5: Non Coding Transcript Variant, BMP6: 3 Prime UTR Variant, TXNDC5: 3 Prime UTR Variant	(15)
	rs7764128	BLOC1S5-TXNDC5: Non Coding Transcript Variant, TXNDC5: 3 Prime UTR Variant, BMP6: 500B Downstream Variant	
	rs8643	BLOC1S5-TXNDC5: Non Coding Transcript Variant, TXNDC5: 3 Prime UTR Variant	
Schizophrenia	rs1225934	BMP6: Intron Variant	(16)
	rs13873	BLOC1S5-TXNDC5: Intron Variant, TXNDC5: Intron Variant	

## 2 Structure of the TXNDC5 protein

**Figure 1:**
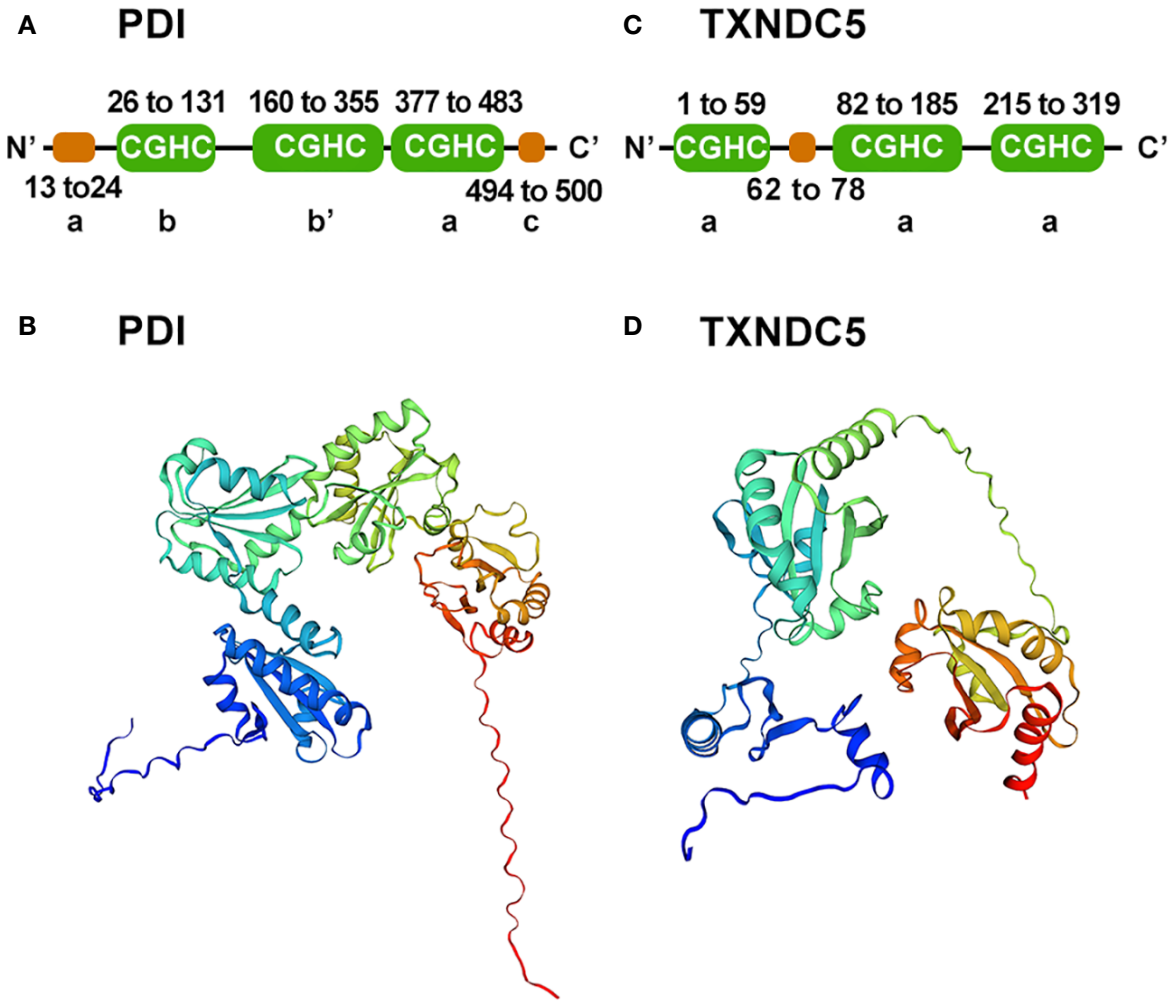
Schematic diagram of the PDI and TXNDC5 structures. **(A)** Prototypical PDI secondary structure domains were analyzed using SMART. PDI consists of four Trx-like domain (a, b, b', a') and a C-terminal domain(c). **(B)** Prototypical PDI three-tier structure was built using SWISS-MODEL. **(C)** TXNDC5 secondary structure domains were analyzed using SMART. TXNDC5 contains there Trx-like domain(a). **(D)** TXNDC5 three-tier structure was built using SWISS-MODEL.

TXNDC5 is a special member of the PDI family. 40 years ago, it was the first-discovered dithiol-disulfide oxidoreductase, and it is capable of reducing, oxidizing and isomerizing disulfide bonds (17). PDI family proteins consist of  four Trx-like structural domains (a, b, b′ and a′) located at the N-terminus and an additional α-helix c structural domain at the C-terminus (18). Together, they form a highly conserved U-shaped structure (19), with each domain connected by an unusually long flexible loop (20). The a and a′ structural domains are redox active due to the Cys-Gly-His-Cys motif and can act synergistically to promote the formation of natural disulfide bonds through the two oxidation sites (20). The b and b′ structural domains lack the Cys-Gly-His-Cys motif, leading to loss of redox activity. However, they are the main substrate binding sites (21–23) and can bind to different substrates through conformational changes (22, 24, 25) (**Figures 1A, B**). Unlike a prototypical PDI protein, TXNDC5 is a rare PDI containing the conserved APWCGHC thioredoxin domain but no b-structural domain (2, 18, 26). The C-terminus of TXNDC5 protein has an endoplasmic reticulum retention signal (KDEL), which is responsible for protein localization to the endoplasmic reticulum. TXNDC5 consists of three redox-like Trx domains (Trx1, Trx2 and Trx3), which form a clover-like structure (25). Each isolated Trx-like structural domain can rapidly import disulfide bonds independently and in a disorderly manner at the same rate as native TXNDC5 without selectivity (18, 27) (**Figures 1C, D**). Each Trx domain contains a CGHC motif as the catalytic domain for PDI activity. In contrast, PDI introduces natural disulfide bonds in an orderly manner through the synergistic action of two redox active sites and selectively proofreads unnatural disulfide bonds. TXNDC5 promote the formation and folding of disulfide bonds through the CxxC motif to enhance protein stability (6, 28). Moreover, TXNDC5 generates H_2_O_2_ through interaction with PDI endoplasmic reticulum oxidoreductase 1α (Ero1α) to participate in the catalytic oxidation reaction between peroxisomal protein 4 (prx4) and TXNDC5, thereby accelerating protein folding (29). Prx4 is typical of the 2-Cys Prx family (30, 31) and forms a homodecamer within which each dimer constitutes a key functional unit (32, 33). Sato Y et al. also demonstrated that TXNDC5 and other PDI family members can collaborate in peroxiredoxin 4-driven oxidative protein folding to increase the rate and fidelity of oxidized protein folding (27).

## 3 TXNDC5 is involved in the inflammatory response

Sepsis is a fatal immune disorder (34, 35), excessive immune responses often result in systemic hypoperfusion, tissue hypoxia, and ultimately organ dysfunction (36, 37). Sepsis triggers the production of multiple pro-inflammatory and anti-inflammatory factors (38). Increased release of pro-inflammatory cytokines leads to dysregulated immune responses (39). Multiple pro- and anti-inflammatory factors including TNF-α, IL-1, IL-6 IL-8, IL-12, interferon (INF)-γ, granulocyte colony-stimulating factor (G-CSF), and the anti-inflammatory cytokine IL-10. Among them, TNF-α and IL-1 are considered to be the main pro-inflammatory factors (40–42). TNF-α activates inflammatory cytokines encoded by the NF-κB signaling pathway, adhesion molecules, gene expression of prostaglandin-synthesizing pathway enzymes, and induction of nitric oxide synthase (iNOS), which activates endothelial cells and leukocytes and exacerbates inflammatory responses (43–47). TXNDC5 was found to be upregulated in lipopolysaccharide (LPS) induced sepsis. Further research has found that inhibition of TXNDC5 attenuated-induced sepsis by suppressing the NF-κB signaling pathway. Moreover, knockdown of TXNDC5 effectively inhibited LPS-induced upregulation of pro-inflammatory cytokines (TNFα, IFN-γ, IL-12, IL-6, and MCP-1) and facilitated the production of the anti-inflammatory cytokine IL-10 (48).

In the development of rheumatoid arthritis (RA), TNF-α, IL-1, IL-6, IL-2 and many other inflammatory factors mediate the inflammatory response (49–52). TXNDC5 can also regulate a variety of cytokines to promote the development of the disease. Wang et al. found that in the presence of LPS, the NF-κB signaling pathway was activated by various pro-inflammatory factors such as IL-6, IL-8 and TNF-α, then induced the expression of TXNDC5, in turn, high expression of TXNDC5 can promote the production of pro-inflammatory factors such as IL-6, IL-8 and TNF-α (11); miR-573 can alleviate inflammation by enhancing the expression of TXNDC5, which in turn inhibits the expression of factors such as toll like receptor 2 (TLR2) and epidermal growth factor receptor (EGFR) (53). Highly expressed TXNDC5 can promote RA by inhibiting C-X-C motif chemokine ligand 10 (CXCL10) (54).

Inflammation often induces fibroblast recruitment and fibrosis, and several inflammatory factors, including TGF-β, IL-13, CD4, have been identified as triggers of fibrosis (55). For example, the pro-inflammatory cytokine interleukin 17A (IL-17A) induces fibrosis in the lungs, liver, kidneys, heart, and skin (56–61). IL-13 selectively induces and activates TGF-β in macrophages to promote fibrosis and promotes fibrosis independently of TGF-β by directly targeting stromal and parenchymal cells (62–64). TXNDC5 plays a critical role of endoplasmic reticulum protein disulfide isomerase (PDI) activity in TGFβ- mediated tissue fibrosis. TGFβ upregulates TXNDC5 by increasing ER stress levels and activating transcription factor 6 (ATF6)-mediated transcriptional regulation. Increased TXNDC5 lead to organ fibrosis by promoting myofibroblasts activation and excessive accumulation of extracellular matrix (ECM) proteins. Highly expressed TXNDC5 contributes to cardiac fibrosis (CF) by promoting ECM protein folding (65). Upregulated TXNDC5 can enhance TGFβ1 signaling by promoting the folding and stabilization of TGFBR1 in lung, leads to pulmonary fibrosis (PF) (66). TXNDC5 triggers renal fibrosis (RF) through enhancing TGF-β signaling pathway in renal fibroblasts (67). TXNDC5 promotes hepatic stellate cell activity and ECM through JNK and STAT3 signaling, thereby causing liver fibrosis (LF) (68). In conclusion, the TGFβ-ATF6-TXNDC5 signaling axis highlights the role of TXNDC5 in fiber formation during the development of fibrosis in heart, lung, kidney and liver organs (69).

TXNDC5 is involved in the tumorigenesis and progression by participating in inflammatory response. Multiple studies demonstrates that inflammation is closely relevant to the onset and progression of cancers (70–73). Specifically, chronic inflammation is involved in immunosuppression, acute inflammation induces cancer cell death via antitumor immunity, and the inflammatory response is also involved in anticancer therapies (74–76). For instance, TNF-α and IL-1β can play an important role in the occurrence of colorectal cancer through increasing the Toll-IL-1 receptor signaling (77). IL-17 produced by γδ T cells plays a key role in breast cancer metastasis (78). Additionally, TXNDC5 promotes cancer progression by regulating various inflammatory factors. TXNDC5 induces rhabdomyosarcoma proliferation survival and migration by regulating interleukin-24 (IL-24) (79), which has a wide range of anticancer activities and gradually be used in clinical therapy (80–82). TXNDC5 can also contributes to abnormal angiogenesis in cervical cancer by regulating inflammatory factor receptor expression of SERPINF1 and TRAF1, which can activate the NF-κB signaling in inflammatory environments (12, 83, 84). In conclusion, the insights have the potential to open new avenues in cancer treatment by targeting TXNDC5 to control aberrant inflammatory responses.

**Figure 2:**
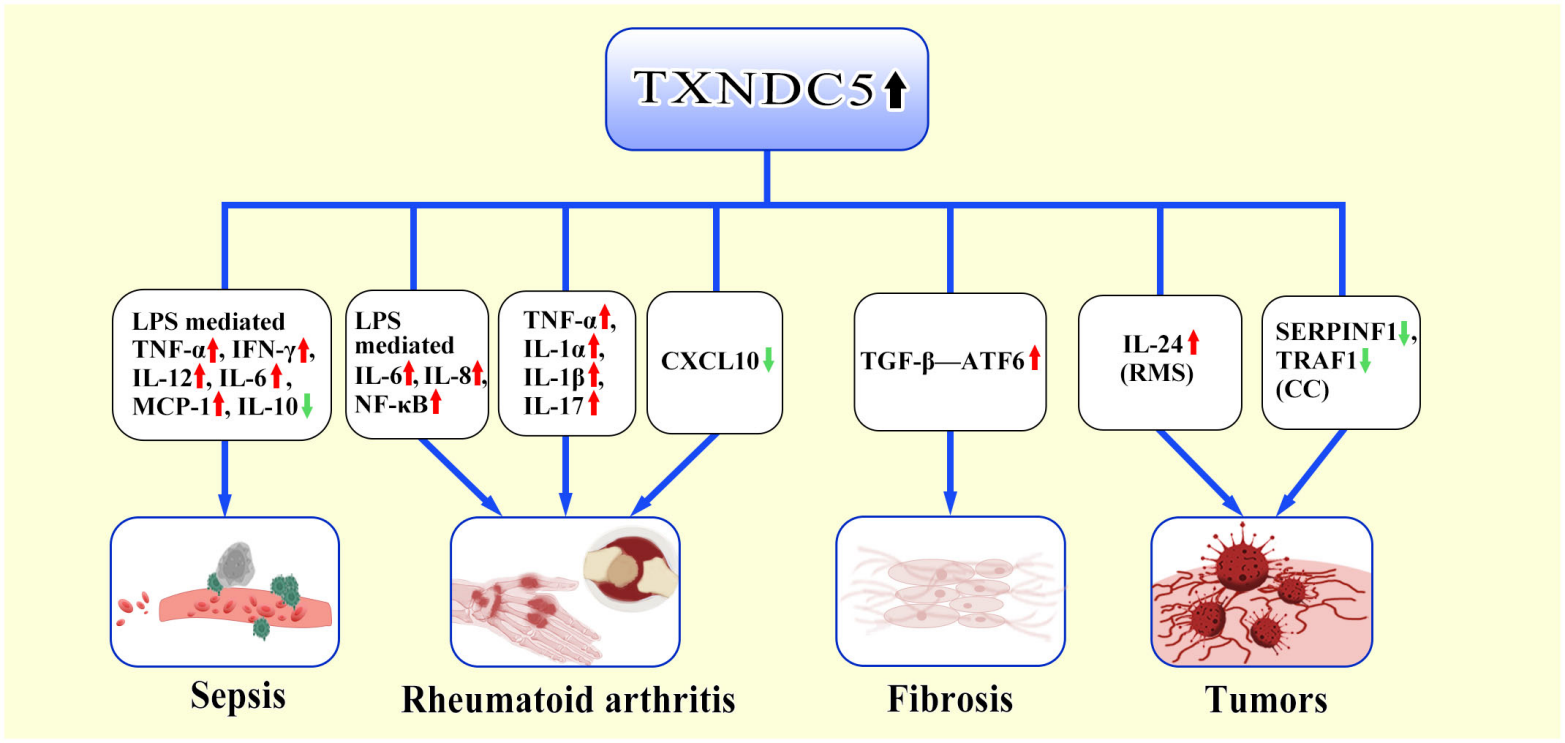
TXNDC5-mediated inflammatory response. TXNDC5 involves in multiple disease by regulating multiple inflammatory factors and inflammatory factor receptors. In sepsis, highly expressed TXNDC5 upregulates pro-inflammatory factors (TNF-α, IFN-γ, IL-12, IL-6, MCP-1) via LPS and downregulates the production of anti-inflammatory factors (IL-10). In RA, TXNDC5 exacerbates RA through LPS-mediated induction of pro-inflammatory factors (IL-6, IL-8, NF-κB); Highly expressed TXNDC5 can upregulate pro-inflammatory factors (TNF-α,IL-1α, IL-1β,IL-17) to promote RA development; Increased TXNDC5 can promote the occurrence of RA by inhibiting CXCL10. In the progress of organ fibrosis, TXNDC5 can mediate the TGFβ-ATF6-TXNDC5 signaling axis to induce multiorgan fibrosis. In tumors, TXNDC5 promotes proliferation of rhabdomyosarcoma (RMS) by inducing IL-24; TXNDC5 contributes to cervical carcinoma (CC) progression by decreasing the expression of SERPINF1 and TRAF1.

In conclusion, TXNDC5 directly or indirectly regulates inflammatory factors and promotes inflammatory responses (**Figure 2**).

## 4 The role of TXNDC5 in rheumatoid arthritis pathogenesis

RA is a systemic autoimmune disease characterized by the proliferation of synovial fibroblasts (SFs), which produce a variety of proteases and inflammatory factors that destroy bone and cartilage (85). In the early stages of RA, the immune system is activated and immune cells (Dendritic cells, T cells and B cells) (86, 87) infiltrate joint tissues, leading to intra-articular hyperplasia and thus inducing synovial hypoxia and hypoperfusion (88). Furthermore, hypoxia induces the overexpression of TXNDC5. Chang et al. found that the expression of TXNDC5 is high in the synovial tissue and blood of RA patients by immunohistochemistry and western blotting (13, 89). Nine SNPs located in the TXNDC5 gene (rs1225936, rs1225938, rs2743992, rs372578, rs408014, rs41302895, rs443861, rs9392189, rs9505298) were found to be closely associated with RA susceptibility (14). Subsequently, Wang et al. found that hypoxia induced TXNDC5 overexpression in the synovial tissues of RA patients, which stimulated synovial fibroblasts to produce adiponectin (ADP). ADP subsequently stimulated synovial fibroblasts secrete cytokines and chemokines to promote inflammation, leading to RA (90). Through further studies, Wang et al. found that increased expression of TXNDC5, toll like receptor 2 (TLR2) and epidermal growth factor receptor (EGFR) could be suppressed by enhancing miR-573 expression during TXNDC5-induced RA, thereby alleviating inflammation (53). Meanwhile, Wang et al. revealed that the expression of TXNDC5 can be upregulated in response to inflammatory factors (LPS, TNF-α and IL-6) and under the control of NF-κB signaling. They found that heat shock cognate 70 protein (HSC70) forms a complex with TXNDC5 in the cytoplasm and their directly interaction can be strengthened in the presence of LPS, TNF-α and IL-6. Further research indicated that LPS stimulation is a key point in IκBβ nuclear translocation and subsequent NF-κB activation. HSC70 activates NF-κB signaling by destabilizing IκBβ protein in the absence of LPS or promoting its nuclear translocation in the presence of LPS. In the nucleus, newly synthesized IκBβ is in a quiescent statement and the NF-κB signaling is activated. Thus, TXNDC5 plays a pro-inflammatory role in RASFs by potentiating the effects of HSC70/IκBβ-mediated NF-κB signaling (11). Thus, the TXNDC5/HSC70-mediated inflammatory pathway forms a vicious circle in the progression of RA, and the two complement each other to play an important role in the RA process. Wang et al. also found that TXNDC5 could promote RA by upregulating TNF-α, IL-1α, IL-1β, and IL-17 (90). Xu et al. found that TXNDC5 overexpression inhibited CXCL10 and tumor necrosis factor-related apoptosis-inducing ligand TNF superfamily member 10 (TRAIL) expression, further contributing to the abnormal proliferation, apoptosis and angiogenesis of RASFs (54). Li et al. asserted that TXNDC5 induces insulin resistance and increases the risk of diabetes mellitus (DM) by inhibiting the expression of insulin-like growth factor binding protein-1 (IGFBP1) (91). The onset and progression of DM are closely related to systemic inflammation and insulin resistance, which is a state of impaired glucose metabolism and insulin dysfunction (92). It was reported that most patients with RA are insulin resistant (93–97). These studies suggested that there is a close connection between RA and DM. The study conducted by Alexander et al. revealed that the levels of fasting glucose are increased in 9 individuals with loss-of-function (LOF) variation in TXNDC5, indicating TXNDC5 can be identified as a potential determinant of type 1 diabetes risk (98).

**Figure 3:**
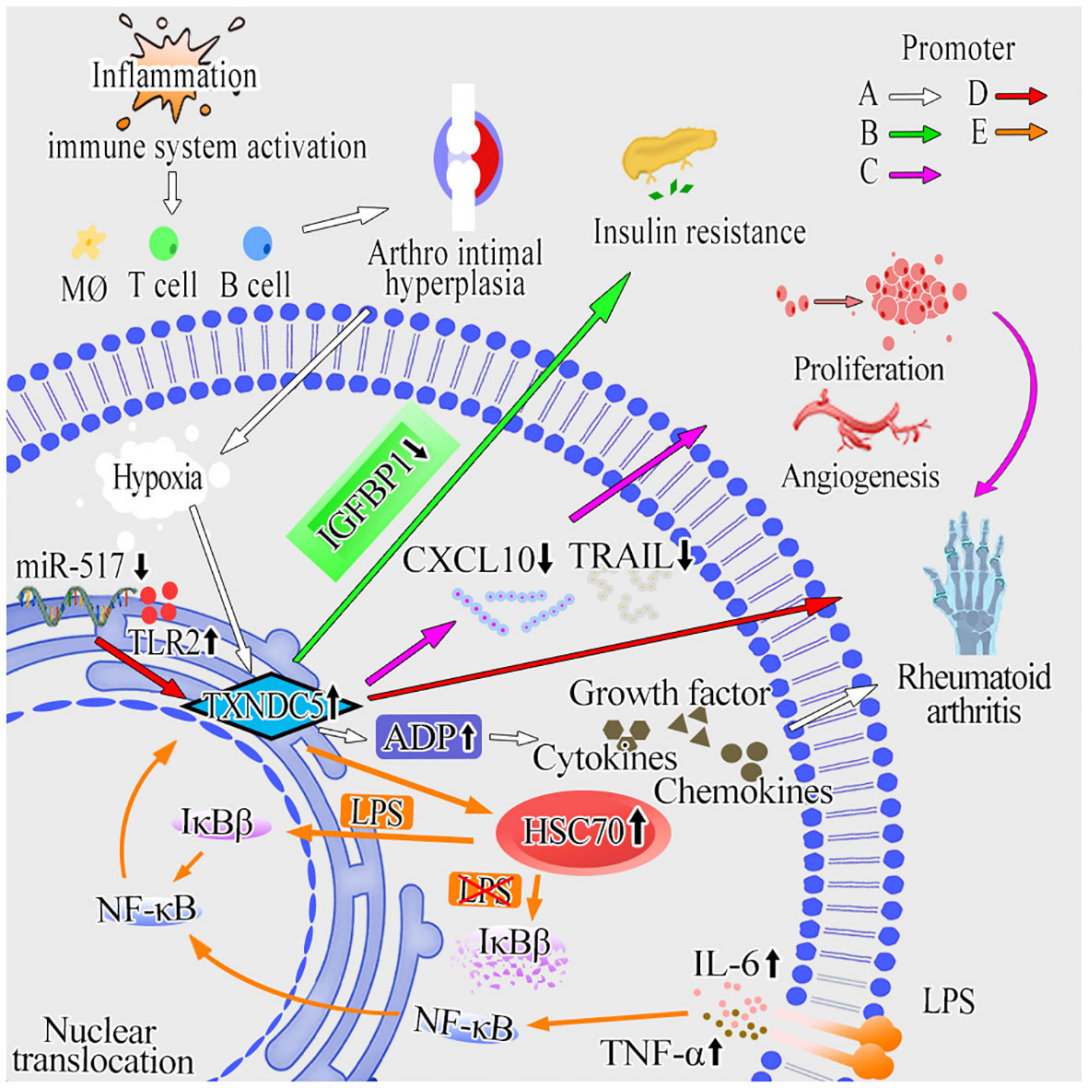
The molecular mechanisms of TXNDC5 promotes RA. **(A)** Inflammation can activate the body’s immune system and contribute to hyperplasia of the joint lining. Hypoxia and hypoperfusion are induced by arthro intimal hyperplasia and increase TXNDC5 expression subsequently. By upregulating ADP, TXNDC5 secretes cytokines, chemokines and growth factors to promote inflammation and RA progression (white arrow). **(B)** TXNDC5 lead to insulin resistance and increase the risk of developing diabetes mellitus by inhibiting the expression of IGFBP1 (green arrow). **(C)** TXNDC5 can inhibit the expression of CXCL10 and TRAIL, leading to abnormal proliferation, apoptosis, and angiogenesis in RASFs (pink arrow). **(D)** Inhibit the expression of miR-573 in RA increased the expression of TXNDC5 and TLR2, which then exacerbates inflammation and induces RA (red arrow). **(E)** The expression of TXNDC5 can be upregulated in response to inflammatory factors (LPS, TNF-α and IL-6) and under the control of NF-κB signaling. In the absence of LPS, HSC70 forms a complex with TXNDC5 in the cytoplasm, and then activates NF-κB signaling by destabilizing IκBβ protein to induce RA; In the presence of LPS, IκBβ can translocate into the nucleus and facilitate the activity of p65, promoting transcription of TXNDC5 and RA progression (orange arrow).

In conclusion, the above studies regarding RA revealed the important facilitating role of TXNDC5 in RA progression and provide a new therapeutic target for the future treatment of RA (**Figure 3**).

## 5 The role of TXNDC5 in the pathogenesis of organ fibrosis diseases and its underlying mechanism

A growing body of data suggests a strong link between TXNDC5 and fibrotic diseases. TXNDC5 was found to be highly expressed in multiple fibrotic diseases, and TXNDC5 is a key pathogenic factor in multiple organ fibrotic diseases. In 2018, Shih et al. used RNA sequencing and gene co-expression network analysis to analyze data from failing human hearts, and found that TXNDC5 was highly upregulated in failing human left ventricular (LVs) (65). Highly expressed TXNDC5 can promote ECM enrichment to induce myocardial fibrosis by increasing NOX4-derived ROS and activating redox-sensitive JNK signaling. CF can lead to cardiac structural and functional remodeling, triggering diastolic dysfunction (99, 100) and consequently heart failure (HF). Increased levels of TXNDC5 expression further enhance the excessive accumulation of myofibroblasts and ECM proteins, leading to CF (65). Suppress the expression of TXNDC5 therefore provides a new therapeutic target for the treatment of CF, in contrast to traditional therapeutic modalities, including angiotensin-converting enzyme inhibitors (ACEI), angiotensin receptor blockers (ARBs) and mineralocorticoid receptor antagonist (MRA) (101–103). Targeting TXNDC5 does not limit the slowing of CF due to lower blood pressure. Moreover, inhibiting TXNDC5 expression can limit CF progression by silencing TGF-β1, thus attenuating fibroblast activation, ECM enrichment (65, 104, 105) and evading hepatotoxicity (106). In summary, silencing TXNDC5 provides new therapeutic strategies to alleviate CF and prevent HF.

In 2020, Lee et al. found that TXNDC5 was highly upregulated in lung tissue from patients with idiopathic pulmonary fibrosis and a bleomycin (BLM)-induced PF mouse models (66). TGF-β and TGFBR2 binding activates the TGFBR1/ER stress/ATF6 transcriptional pathway to drive TXNDC5 enrichment in lung fibroblasts, which in turn induces fibroblast hyperactivation, proliferation, and ECM enrichment through activation of TGF-β classical (SMAD3) (107) and nonclassical (JNK, ERK, PI3K, p38, MAPK) signaling (108), leading to PF. Suppress the TGF-β pathway represents an attractive approach to treat pulmonary fibrosis, but extensive inhibition of TGF-β leads to hepatotoxicity (109) and cardiotoxicity (110, 111); targeting knockdown of TGF-β1 causes interstitial pneumonia and systemic perivascular inflammation (112, 113); and targeting inhibition of TGFBR1 promotes impaired alveolar and epithelial cell production (114). TGF-β expression is necessary for lung organogenesis and homeostasis *in vivo* (115), therefore, direct down regulation of TGF-β rarely reaches the early clinical trial stage (109). However, compared with TGF-β, inhibit the expression of TXNDC5 showed no significant adverse effects.

Chen et al. Microarray data from renal biopsy specimens from CKD patients were analyzed and increased renal TXNDC5 expression was verified using gene knockout, flow cytometry, and immunohistochemistry. This study experimentally hypothesized to be under the control of the TGF-β1/ATF6/TXNDC5/TGFBR1 signaling axis, resulting in the enhancement of the folding and stability of TGFBR1. The signaling pathway leads to the amplification of TGF-β1 signaling and a series of renal fibrotic responses (67). Studies have shown that inflammation, tubular injury and other factors increase pro-fibrotic and inflammatory factors, inflammatory cells in large amounts, including TGF-β and macrophages (116). TGF-β is a key factor in the development of RF (117). Increasing expression of TGF-β drives fibroblast activation into collagen-secreting myofibroblasts (118–120), characterized by α-smooth muscle actin (αSMA) expression and excessive ECM deposition, leading to abnormal renal structure (121). In the process of treating RF, Chen et al. found that TXNDC5 deletion effectively ameliorated the development and progression of RF induced by various injuries in mice (67), providing a new and effective method for treating RF.

Hung et al. studied liver fibrosis (LF) and validated that the TGFβ/ATF6/TXNDC5/JNK/STAT3 signaling axis, suggesting that TXNDC5 plays a key role in the formation of LF (68). LF is generally caused by chronic liver injury (122), such as viral infections, nonalcoholic steatohepatitis (NASH), alcohol consumption (AC) and biliary obstructive disease (123, 124). Chronic hepatocellular injury could lead to epithelial/endothelial barrier damage, the release of inflammatory cytokines and the agglomeration of inflammatory cells followed by the secretion of profibrotic cytokines. Hepatic stellate cells (HSC) are activated and transformed into myofibroblasts, which leading to ECM enrichment, fibrous septa formation and regenerative nodules (124, 125). The activation of hepatic stellate cells into myofibroblast-like cells is the central link in the development of LF (126). Therefore, therapies that reduce HSC activation and ECM accumulation have become the mainstay of treatment for LF. TXNDC5 activates HSC through reactive oxygen species (ROS)-dependent JNK signaling; TXNDC5 also enables HSCs to avoid apoptosis via STAT3 signaling, leading to the enrichment of activated HSCs and excessive fibrotic in the liver. Inhibition of the catalytic function of TXNDC5 abrogates JNK and STAT3 activation, leading to downstream fibrotic responses (68). Silencing TXNDC5 reduces liver fibrosis in mice (127). Targeting TXNDC5 in HSCs reduces LF through limiting HSC cell activation by inhibition of noncanonical TGF-β signaling.

**Figure 4:**
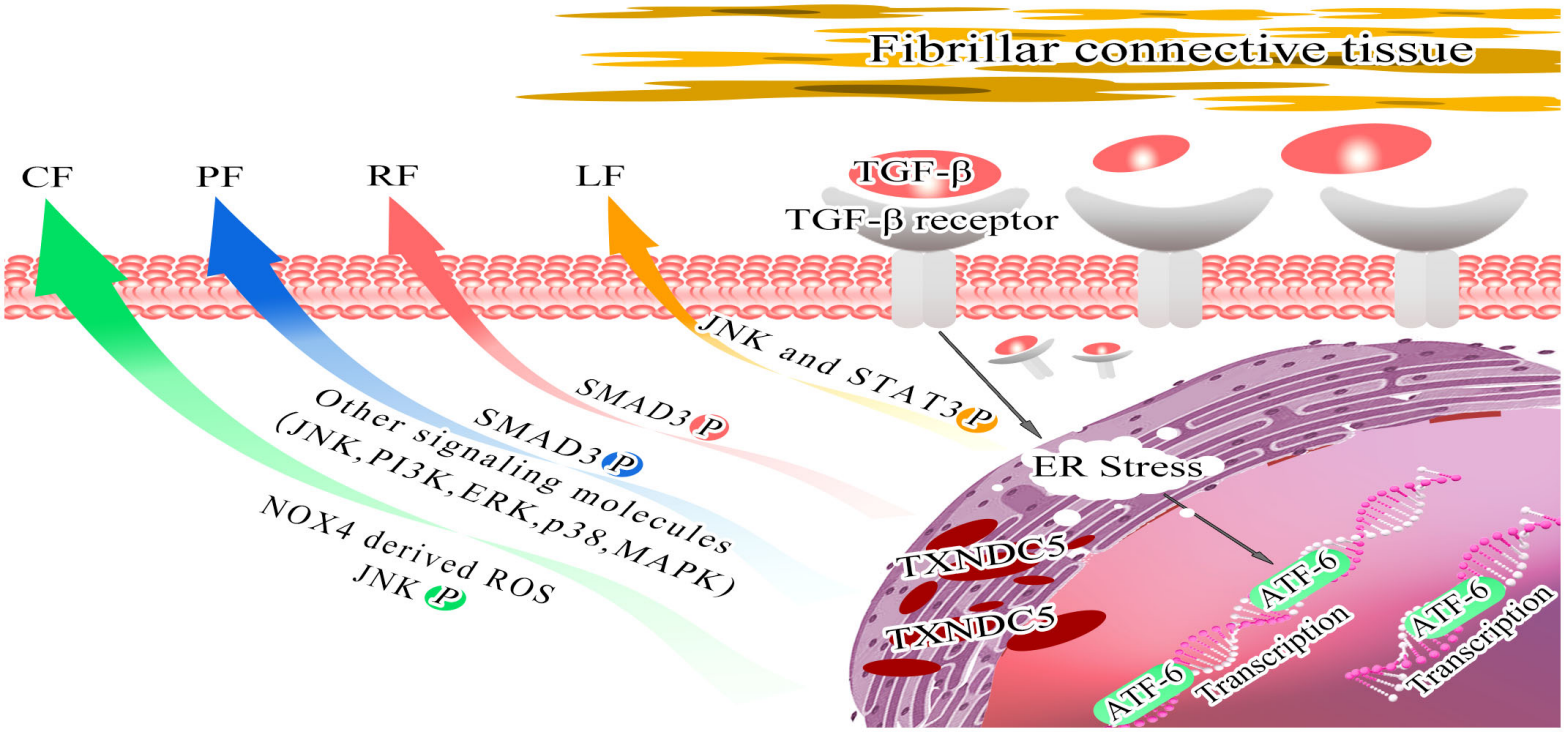
Model depicting the role of TXNDC5 promote multiorgan fibrosis. Stimulation of endoplasmic reticulum stress by TGFβ activates ATF6-mediated transcriptional branching, contributing to the upregulation of TXNDC5 expression, which mediates downstream effector molecules to induce organ fibrosis. Increased TXNDC5 expression induces CF by increasing NOX4-derived ROS and activating redox-sensitive JNK signaling (green arrow); leads to PF by activating SMAD3 phosphorylation and inducing other signaling molecules (JNK, PI3K, ERK, p38 and MAPK) (blue arrow); leads to RF by activating SMAD3 signaling (red arrow); and leads to LF by activating JNK signaling and STAT3 signaling (orange arrow).

In summary, TXNDC5 is associated with key factors that promote the development of organ fibrosis. Targeting TXNDC5 deletion may be a potential new therapeutic strategy to improve fibrotic disease (**Figure 4**, **Table 2**).

**Table 2:** TXNDC5 promotes the organ fibrosis signaling pathway.

Organ Fibrosis	Disease Abbreviation	TXNDC5 Upstream adjustment pathway	Downstream effectors	Results	Reference
Cardiac fibrosis	CF	TGFβ/ER stress/ATF6 mediates transcription	1.NOX4-derived ROS 2. Phosphorylated JNK signaling	Fibroblast activation and excessive accumulation of ECM proteins contribute to CF	(65)
Pulmonary fibrosis	PF	TGFβ binding to TGFBR2 activates TGFBR1/ER stress/ATF6-mediated transcription	1. SMAD signal 2. Other signaling molecules (JNK, ERK, PI3K, p38, MAPK)	Fibroblast activation and excessive accumulation of ECM proteins contribute to PE	(66)
Renal fibrosis	RF	TGFβ binding to TGFBR2 activates TGFBR1/ER stress/ATF6-mediated transcription	1. Redox-sensitive SMAD3 signaling	Fibroblast activation and excessive accumulation of ECM proteins contribute to RF	(67)
Liver Fibrosis	LF	TGFβ/ER stress/ATF6 mediates transcription	1. Redox-sensitive JNK signaling and STAT3 signaling	Fibroblast activation and excessive accumulation of ECM proteins contribute to LF	(68)

## 6 TXNDC5 and tumor tissues

### 6.1 TXNDC5 is highly expressed in a variety of tumor tissues

Increasing evidence has revealed that TXNDC5 and tumors progression are closely related (128). Several studies found that TXNDC5 showed significantly increased expression in a variety of cancer tissues. Chang et al. found that TXNDC5 was significantly expressed in tumor tissues, including invasive ductal carcinoma of the breast, squamous cell carcinoma of the cervix, squamous cell carcinoma of the esophagus, papillary plasmacytoma of the ovary, and prostate cancer (13). It was reported that TXNDC5 was also found to have procarcinogenic effects in tissues of several cancers, including prostate cancer (PCa) (129), colorectal cancer (CRC) (130) (127), lung cancer (LCA) (131), non-small cell lung cancer (NSCLC) (132), ovarian cancer (OC), gastric cancer (GC) (133, 134), cervical cancer (CC) (12), esophageal squamous cell carcinoma (ESCC) (135), and hepatocellular carcinoma (HCC) (136). In samples from patients with LCA, TXNDC5 protein expression was upregulated in more than 60% of NSCLC tissues (137). Batool et al. found that the increased expression of TXNDC5 was mostly due to increased levels of transcription and translation of the *TXNDC5*, especially the increased transcription of the *TXNDC5*, which was also found in tissues obtained from patients in the early stages of colorectal cancer (130). In addition, several experiments have demonstrated that TXNDC5 is overexpressed in colorectal cancer tissues, revealing that TXNDC5 is a tumor-enhancing gene that promotes the proliferation and migration of a variety of tumor cells. By immunohistochemical studies, Wu et al. found that TXNDC5 was highly expressed in gastric cancer cells, particularly in hypofractionated adenocarcinoma (133). Regarding hepatocellular carcinoma tissue, TXNDC5 expression is increased in poorly differentiated hepatocellular carcinomas but not in highly differentiated tumors. In Pca, TXNDC5 was significantly overexpressed in androgen-intrinsic prostate cancer and desmoplastic-resistant prostate cancer (129). In ESCC, Wang et al. found that TXNDC5 showed highly expression, indicating that ESCC with high TXNDC5 expression had a poor prognosis (135). By using in silico analysis, Kocatürk et al. found that the expression pattern of TXNDC5 family members is different between tumor tissues and healthy tissues, and the expression of TXNDC5 is proportional to the grades of diffuse glioma tumors (138). In summary, TXNDC5 is a typical cancer-enhancing gene that is highly expressed and overexpressed in tumor tissues of several cancers and plays an important role in the development of cancer.

### 6.2 The regulatory mechanism of TXNDC5 in cancer development

#### 6.2.1 Hypoxia induces high expression of TXNDC5

TXNDC5, like most members of the PDI family, is involved in the correct folding and formation of disulfide bonds in newly synthesized proteins through disulfide isomerase and chaperone protein activity, and plays an important role in prevention of endoplasmic reticulum stress (5–7). Sullivan et al. found that PDI was needed for endothelial cell survival under both normoxic and hypoxic conditions, but TXNDC5 was only highly expressed and exerted a protective effect on endothelial cells under hypoxic conditions. Tan et al. found that hypoxia could induce upregulation of TXNDC5 in colorectal cancer tissues by elevating the expression of hypoxia-inducible factor-1α (HIF-1α), leading to reduced ROS production (130). It was previously reported that ROS is directly or indirectly involved in endoplasmic reticulum homeostasis and protein folding, thereby triggering endoplasmic reticulum stress and possibly inducing apoptosis in response to excessive endoplasmic reticulum stress (139). Hypoxia can inhibit hypoxia-induced ROS/ER stress signaling and promote proliferation and clone formation in colorectal cancer cells by inducing TXNDC5 overexpression through the upregulation of HIF-1α (130). Wang et al. observed that TXNDC5 was upregulated in prostate cancer cells after prolonged androgen deprivation therapy (ADT) due to ADT-induced hypoxia upregulating TXNDC5 expression through androgen receptor (AR) protein signaling, thereby enhancing their interaction, stability and transcriptional activity. This mechanism further regulates TXNDC5 expression through HIF-1α and miR-200b-dependent pathways (129). The above results suggest that TXNDC5 may play a role as a hypoxia-induced stress survival factor in tumor cells, contributing to tumor cell growth and proliferation under hypoxic conditions.

#### 6.2.2 The diverse oncogenic mechanism of TXNDC5 in various cancers

Numerous studies have shown that increased TXNDC5 expression is regulated by multiple factors. In 2017, Xu et al. demonstrated that TXNDC5 is a susceptibility gene in cervical cancer using Taqman genotype. They point out that TXNDC5 is highly expressed in cervical squamous cancer tissues. TXNDC5 can promote angiogenesis, angiogenic mimicry and cell metastasis in cervical cancer (12). Du et al. identified TXNDC5 as a target of MELLT3 mediated m6A modification by MeRIP-seq, and confirmed the positive correlation between TXNDC5 and METTL3 at the protein and RNA levels. Their further study found that the m6A readers (YTHDF2 and IGF2BP2/3) could interact with TXNDC5 mRNA. Mechanically, IGF2BP2/3 enhanced TXNDC5 mRNA stability, whereas YTHDF2 may promote TXNDC5 mRNA degradation. Thus, METTL3 promotes proliferation and metastasis of CC cells by upregulating TXNDC5 expression via m6A-reader-dependent way (140). Yu et al. demonstrated that circRNA-104718 acts as a competitive endogenous RNA for miR-218-5p to regulate TXNDC5 in HCC, and thus promote HCC (141). The next year, Zang et al. detected significantly elevated protein levels of TXNDC5 in HCC tissues and cells by western blotting, and found that circ_0000517 could promote TXNDC5 overexpression by inhibiting miR-1296-5p. Further research shown that TXNDC5 overexpression could enhance HCC cell viability, promote HCC cell colony formation, shorten cell cycle, and promote cell proliferation and migration (136). Wang et al. confirmed that HERG1 induces to poor prognosis in esophageal squamous cell carcinoma (ESCC) patients by promoting cell proliferation, migration, and invasion, while these effects can be reversed by altering the expression of TXNDC5 and its downstream PI3K/AKT pathway. The study suggested that TXNDC5 is a key point in the pathway of HERG1 promotes tumor progression (135). Ge et al. revealed that the TBX15/TXNDC5 axis play a crucial role in the genesis and progression of glioma; TBX15 exerts its oncogenic roles by regulating transcriptional activation of TXDNC5 (142). Overall, these results indicate that TXNDC5 is affected by different regulators in various cancers and that TXNDC5 plays an important role in promoting cancer proliferation, invasion and metastasis.

TXNDC5 also influenced by other factors that promote the development of cancer. For example, TXNDC5 expression is induced by three endoplasmic reticulum stress conditions, including glucose deprivation, serum deprivation and the presence of tunicamycin (TM) (143, 144), endoplasmic reticulum stress is a key factor in tumor-promoting mechanisms (144), which affect protein glycosylation and ATP production, leading to endoplasmic reticulum stress and the accumulation of unfolded or misfolded proteins. In clear cell renal cell carcinoma (ccRCC), ccRCC cells adapt to this stressful environment and escape apoptosis (143). In pancreatic cancer, NR4A1 (Nur77, TR3) regulates TXNDC5 expression, maintains low levels of stress by ROS in cancer cells, and promotes pancreatic cancer cell proliferation (145). Chawsheen et al. demonstrated that TXNDC5 interacts with sulfiredoxin (Srx) through IP experiments and that the two together maintain endoplasmic reticulum homeostasis in human lung cancer cells, thereby promoting cell colony formation and migration (131). Charlton et al. found that TXNDC5 inhibited the lipocalin signal pathway by interacting with AdipoR1 in HeLa cells (9). Regarding to renal cell carcinoma (RCC), the ratio of TXNDC5/AdipoR1 expression was significantly higher in metastatic renal cell carcinoma tissues than in nonmetastatic controls (146). The presence of TXNDC5 in metastatic renal cell carcinoma promotes cell growth, migration, invasion and increases resistance of cancer cells to chemotherapeutic agents (143). However, there is not sufficient evidence for a stable interaction between AdipoR1 and TXNDC5 (146). Moreover, it is possible that the interaction between AdipoR1 and TXNDC5 is regulated by various interaction factors and therefore varies due to the characteristics of the interaction factors. It is debatable whether the tumorigenic properties of TXNDC5 expression in RCC cells are related to the inhibitory regulation of lipocalin tumor suppressor signaling.

**Figure 5:**
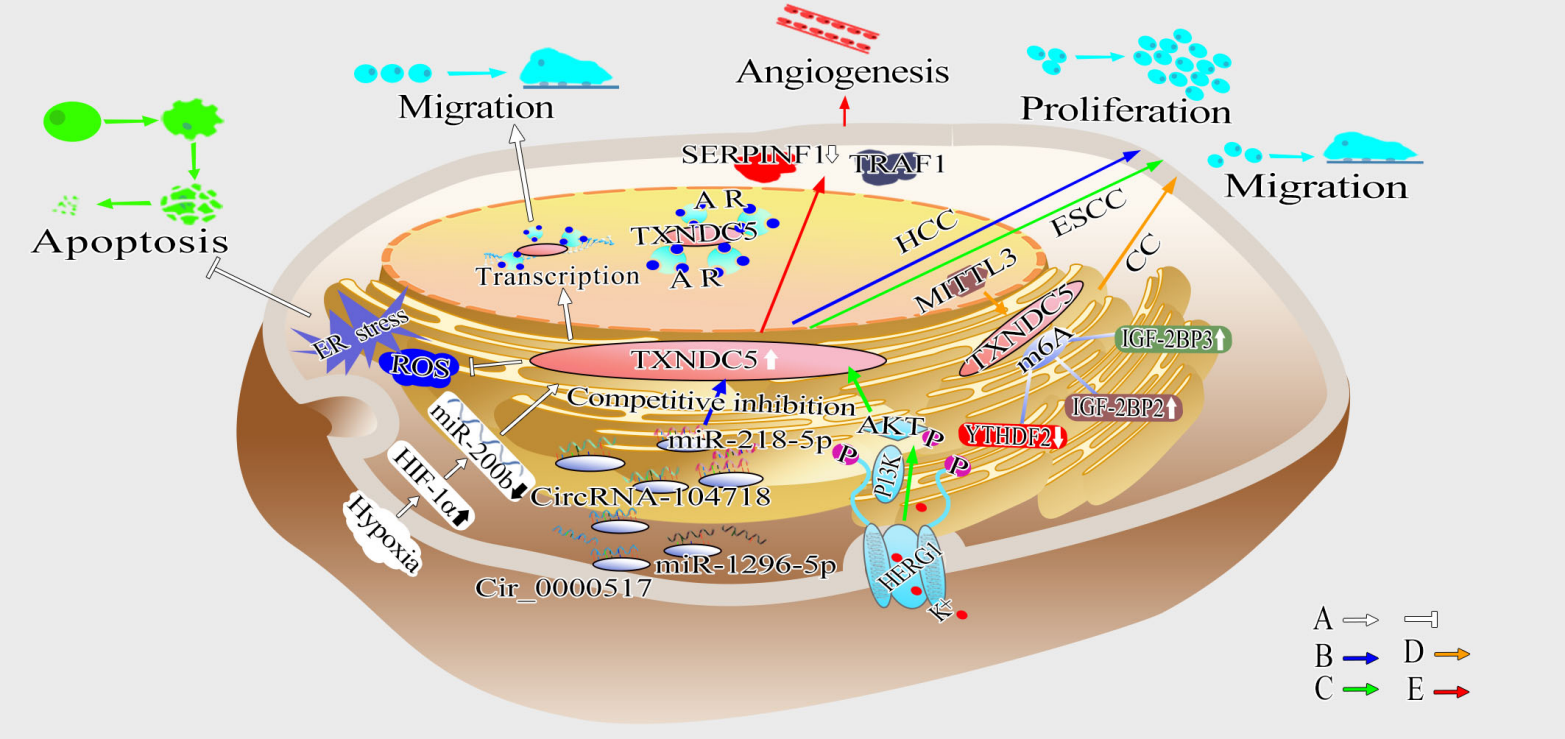
Schematic diagram showing the mechanism by which TXNDC5 promotes cancer. **(A)** TXNDC5 can be upregulated under hypoxia and play a crucial role in anti-apoptosis by regulating hypoxia-induced ROS/ER stress signaling; Hypoxia induce the expression of TXNDC5 through HIF-1α in an miR-200b-dependent manner, and strengthen the interaction between TXNDC5 and AR to promote proliferation and migration (white arrow). **(B)** Circ_0000517 and circRNA-104718 acted as miR-1296-5p and miR-218-5p competitive endogenous RNAs, respectively, upregulating TXNDC5 expression to promote HCC proliferation and metastasis (blue arrow). **(C)** HERG1 leads to ESCC proliferation, migration and invasion through TXNDC5 by mediating the PI3K/AKT signaling pathway (green arrow). **(D)** METTL3 promotes proliferation and metastasis of cervical cancer (CC) cells by upregulating TXNDC5 expression via m6A-reader-dependent way (orange arrow). **(E)** Highly expressed TXNDC5 can promote angiogenesis by inhibiting the expression of SERPINF1 and TRAF1 (red arrow).

In summary, TXNDC5 has different oncogenic mechanisms in different cancers, and the complex mechanisms of TXNDC5 in cancer tissues deserve further exploration (**Figure 5**).

### 6.3 TXNDC5: a promising potential tumor diagnostic marker or therapeutic target

TXNDC5 can be used as a diagnostic marker and a therapeutic target in cancer. Decrease the expression of TXNDC5 in cancer tissues can attenuate cell viability, inhibit cell colony formation, induce cell cycle arrest and apoptosis. In ccRCC, TXNDC5 can be used as a prognostic criterion for patients (143). Ren et al. also found that the expression of TXNDC5 was negatively correlated with the chemosensitivity of ccRCC, and inhibit the expression of TXNDC5 increased the resistance of ccRCC to chemotherapeutic drugs, reduced the killing of cancer cells by chemotherapeutic drugs, and promoted the development of cancer (143). These results indicated that TXNDC5 probably can be used as a diagnostic and prognostic biomarker, indicating that TXNDC5 is a promising therapeutic target for ccRCC. In gastric adenocarcinoma tissues Wu et al. found that TXNDC5 is highly expressed in gastric cancer cells by immunohistochemistry and its expression is significantly increased in poorly differentiated adenocarcinomas, suggesting that the expression of TXNDC5 is significantly associated with the prognosis of gastric adenocarcinomas at the cardia (133). Nissom et al. identified that TXNDC5 was expressed in HCC but not in highly differentiated HCC, indicated that the expression of TXNDC5 could be used to predict the progression of HCC. In CRC (130) and NSCLC (137), TXNDC5 is highly expressed in the early stages of cancer, so TXNDC5 can be used as a means to diagnose early cancer. Chawsheen et al. revealed that inhibiting the expression of TXNDC5 may reduce the incidence of lung cancer. Further research indicated that the Srx-TXNDC5 complex may be used to predict the survival probability for lung cancer patients and as a therapeutic target or molecular diagnostic tool in human lung cancer pathogenesis (147). In brief, TXNDC5 is a promising prognostic marker for cancer progression, a therapeutic target and a molecular diagnostic indicator for cancer pathogenesis. In conclusion, TXNDC5 plays an essential role in cancer diagnosis and therapy.

## 7 The role of TXNDC5 in other diseases

It is well known that TXNDC5 is regulated by hypoxia (3), which induces vasoconstriction and coronary arteriosclerosis. Camargo et al. reported that TXNDC5 promotes the expression of proangiogenic proteases by regulating AP-1-dependent gene expression and induces angiogenesis in response to TNF-α (8). Yeh et al. revealed that TXNDC5 promotes atherosclerosis *in vivo*. Mechanically, TXNDC5 induces ubiquitination and proteasome-mediated degradation of HSF1, destabilizes eNOS protein by inhibiting HSP90 (148). Their further studies found that TXNDC5 deletion in vascular endothelium result in increased eNOS protein and reduced atherosclerosis in apoE ^−/−^ mice. Meanwhile, Kuhlencordt et al. found that atherosclerosis, aortic aneurysm formation and ischemic heart disease are accelerated in apoE/eNOS double knockout mice (149). These above findings indicates that TXNDC5 may lead to vascular diseases through regulating apoE and eNOS.

Holmgren et al. recently found that loss-of-function mutations in TXNDC5 may prevent key peptides from functioning properly, thus causing insulin hypersecretion (150). This resulted in insufficient insulin secretion. Subsequently, Alexander et al. showed that TXNDC5 could catalyze the reduction of insulin disulfide bonds and weaken the binding activity of insulin to the insulin receptor, thus causing abnormalities in glucose tolerance in the body, revealing an important role for TXNDC5 in promoting the development of diabetes (98). Recently, Li et al. found that high expression of TXNDC5 inhibited the expression of IGFBP1 to induce insulin resistance, increasing the risk of developing DM (91).

Ramírez et al. found that the severity of fatty liver induced by apolipoprotein E (ApoE) knockout was negatively correlated with the levels of TXNDC5 protein and mRNA. Their further study revealed that the expression of TXNDC5 reflected squalene’s anti-lipotropic properties and sensitivity to lipid accumulation (151). Karatas et al. found that four members of the PDI family (TXNDC5, PDIA4, PDIA3 and P4HB) were specifically upregulated in adult ZZ-AATD-mediated liver disease by studying hepatocyte function in patients with ZZ-type α1-antitrypsin deficiency (152). In the mechanism of interferon-stimulated gene 15 (ISG15)-induced hepatitis C virus (HCV) infection, ISG15 may promote HCV replication by regulating TXNDC5 (153–155).

Kim et al. found a correlation between TXNDC5 and skin aging by immunohistochemical assay and qRT−PCR in the skin tissues of 20 patients (156). In addition, TXNDC5 expression was higher in peripheral blood mononuclear cells in young adults than in older adults, suggesting that altered TXNDC5 expression may be associated with endothelial cell apoptosis (157).

In addition, certain SNP loci of TXNDC5 are closely associated with disease susceptibility. In a Korean population, three exonic SNPs (rs1043784, rs7764128 and rs8643) in TXNDC5 were found to be positively associated with nonsegmental vitiligo (NSV) (15). A SNP (rs13873) in the TXNDC5 gene and haplotype rs1225934-rs13873 in BMP6-TXNDC5 play a role in the selective impairment of persistent attention disorder in schizophrenia (16). The TXNDC5-related SNP (rs13196892: TXNDC5 | MUTED) that may be associated with human age was first identified in a study exploring female menopause/age-related factors (158).

In addition, TXNDC5 may be a predictive marker for histone deacetylase inhibitor (HDACI) resistance in cutaneous T-cell lymphoma during drug treatment of the disease (159), a predictive marker for resistance to bortezomib in refractory/relapsed multiple myeloma (160), a regulatory target of simvastatin to enhance docetaxel-induced cytotoxicity in human prostate cancer cells (161) and may be a key factor in the antioxidant effect of statins. Atorvastatin inhibits ROS production and Nox2 activity by promoting the membrane translocation of TXNDC5 in lipid rafts and enhancing the colocalization of TXNDC5 and Nox2, thus exerting antioxidant effects (162). Moreover, direct B lymphocyte LS-TA of TXNDC5 is an early biomarker of vaccine response in novel coronavirus pneumonia (COVID-19) (163).

In summary, TXNDC5 is expected to serve as a target for gene or drug therapy in an effort to change the trajectories of the aforementioned diseases.

## 8 Summary

TXNDC5, a member of the PDI protein family, is mainly found in tissues such as the brain, spleen, lung, liver, kidney, pancreas and testis. TXNDC5 consists of three redox-like Trx structural domains, each of them contains a CGHC motif that acts as an active catalytic structural domain for PDI activity, thereby regulating the rate of disulfide bond formation, isomerization and degradation of target proteins, thus altering the protein conformation and activity to improve protein stability. High expression of TXNDC5 is a key factor in the development of inflammation, cancer, rheumatoid arthritis, organ fibrosis, diabetes and other diseases.

In sepsis, RA and many other inflammatory diseases, TXNDC5 mediates the expression of a variety of inflammatory factors and receptors, which promotes the inflammatory response that leads to disease.

In fibrotic diseases, TXNDC5 acts as a mediator of TGF-ß signaling and amplifies TGF-ß induced fibrotic responses through its PDI activity to induce fibroblast activation, proliferation, and ECM production, inducing fibrosis in multiple organs such as the heart, lungs, kidneys, and liver.

In cancer, the SNPs in TXNDC5 gene are significantly associated with genetic susceptibility to a variety of cancers, including cervical cancer, hepatocellular carcinoma, liver cancer and esophageal cancer. Increased TXNDC5 expression can mediate neovascularization and promote cancer cell proliferation, invasion and metastasis.

In addition, TXNDC5 is strongly associated with a variety of diseases, such as diabetes, fatty liver, schizophrenia, and NSV.

Therefore, targeting TXNDC5 provides a powerful new tool for disease diagnosis and treatment. However, the specific roles and mechanisms of TXNDC5 in different diseases are not yet fully understood, and large-scale clinical trials are needed to validate the mechanism of TXNDC5 in different diseases as well as targeted therapies in the future.

## Author contributions

MJ: Writing – original draft. YZ: Writing – review & editing. XS: Conceptualization, Funding acquisition, Supervision, Writing – review & editing. BX: Conceptualization, Funding acquisition, Supervision, Writing – review & editing.

## Glossary

**Table:**

AC	alcohol consumption
ACEI	angiotensin-converting enzyme inhibitors
ADP	adiponectin
ADT	androgen deprivation therapy
ApoE	apolipoprotein E
AR	androgen receptor
ARBs	angiotensin receptor blockers
ATF6	activating transcription factor 6
CC	cervical cancer
ccRCC	clear cell renal cell carcinoma
CF	cardiac fibrosis
CRC	colorectal cancer
CXCL10	C-X-C motif chemokine ligand 10
DM	diabetes mellitus
ECM	extracellular matrix
EDEM3	alpha -mannosidase–like protein 3
EGFR	epidermal growth factor receptor
Endo-PD	endothelial protein-disulfide isomerase
Ero1α	endoplasmic reticulum oxidoreductase1α
ESCC	esophageal squamous cell carcinoma
G-CSF	granulocyte colony-stimulating factor
GC	gastric cancer
HCC	hepatocellular carcinoma
HCV	hepatitis C virus
HF	heart failure
HDACI	histone deacetylase inhibitor
HERG1	human ether a-go-go-related gene 1
HIF-1α	hypoxia-inducible factor-1α
HSC	Hepatic stellate cells
HSC70	heat shock cognate 70 protein
HSF1	heat shock factor 1
HSPs	heat shock proteins
IGFBP1	insulin-like growth factor binding protein-1
IL-17A	interleukin 17A
Inos	induction of nitric oxide synthase
ISG15	interferon-stimulated gene 15
LCA	lung cancer
LF	liver fibrosis
LOF	loss-of-function
LPS	lipopolysaccharide
LVs	left ventricular
MRA	mineralocorticoid receptor antagonist
NSCLC	non-small cell lung cancer
NSV	nonsegmental vitiligo
OC	ovarian cancer
PCa	prostate cancer
PDI	protein disulfide isomerase
PF	pulmonary fibrosis
prx4	peroxisomal protein 4
RA	rheumatoid arthritis
RCC	renal cell carcinoma
RF	renal fibrosis
SFs	synovial fibroblasts
Srx	sulfiredoxin
TLR2	toll like receptor 2
TM	tunicamycin
TRAIL	TNF superfamily member 10
TNF-α	tumor necrosis factor-α
TXNDC5	Thioredoxin domain containing protein-5.

## References

[1] Knoblach B Keller BO Groenendyk J Aldred S Zheng J Lemire BD. ERp19 and ERp46, new members of the thioredoxin family of endoplasmic reticulum proteins. Mol Cell Proteomics MCP. (2003) 2:1104–19. doi: 10.1074/mcp.M300053-MCP200 12930873

[2] Horna-Terron E Pradilla-Dieste A Sanchez-de-Diego C Osada J. TXNDC5, a newly discovered disulfide isomerase with a key role in cell physiology and pathology. Int J Mol Sci. (2014) 15:23501–18. doi: 10.3390/ijms151223501 PMC4284777 25526565

[3] Sullivan DC Huminiecki L Moore JW Boyle JJ Poulsom R Creamer D. EndoPDI, a novel protein-disulfide isomerase-like protein that is preferentially expressed in endothelial cells acts as a stress survival factor. J Biol Chem. (2003) 278:47079–88. doi: 10.1074/jbc.M308124200 12963716

[4] Edman JC Ellis L Blacher RW Roth RA Rutter WJ. Sequence of protein disulphide isomerase and implications of its relationship to thioredoxin. Nature. (1985) 317:267–70. doi: 10.1038/317267a0 3840230

[5] Freedman RB Hirst TR Tuite MF. Protein disulphide isomerase: building bridges in protein folding. Trends Biochem Sci. (1994) 19:331–6. doi: 10.1016/0968-0004(94)90072-8 7940678

[6] Okada S Matsusaki M Arai K Hidaka Y Inaba K Okumura M. Coupling effects of thiol and urea-type groups for promotion of oxidative protein folding. Chem Commun (Cambridge England). (2019) 55:759–62. doi: 10.1039/C8CC08657E 30506074

[7] Gulerez IE Kozlov G Rosenauer A Gehring K. Structure of the third catalytic domain of the protein disulfide isomerase ERp46. Acta Crystallogr Sect F Struct Biol Cryst Commun. (2012) 68:378–81. doi: 10.1107/S1744309112005866 PMC3325802 22505402

[8] Camargo L Babelova A Mieth A Weigert A Mooz J Rajalingam K. Endo-PDI is required for TNFα-induced angiogenesis. Free Radic Biol Med. (2013) 65:1398–407. doi: 10.1016/j.freeradbiomed.2013.09.028 24103565

[9] Charlton HK Webster J Kruger S Simpson F Richards AA Whitehead JP. ERp46 binds to AdipoR1, but not AdipoR2, and modulates adiponectin signalling. Biochem Biophys Res Commun. (2010) 392:234–9. doi: 10.1016/j.bbrc.2010.01.029 20074551

[10] Yu S Ito S Wada I Hosokawa N. ER-resident protein 46 (ERp46) triggers the mannose-trimming activity of ER degradation-enhancing alpha-mannosidase-like protein 3 (EDEM3). J Biol Chem. (2018) 293:10663–74. doi: 10.1074/jbc.RA118.003129 PMC6036223 29784879

[11] Wang L Dong H Song G Zhang R Pan J Han J. TXNDC5 synergizes with HSC70 to exacerbate the inflammatory phenotype of synovial fibroblasts in rheumatoid arthritis through NF-kappaB signaling. Cell Mol Immunol. (2018) 15:685–96. doi: 10.1038/cmi.2017.20 PMC6123406 28603283

[12] Xu B Li J Liu X Li C Chang X. TXNDC5 is a cervical tumor susceptibility gene that stimulates cell migration, vasculogenic mimicry and angiogenesis by down-regulating SERPINF1 and TRAF1 expression. Oncotarget. (2017) 8:91009–24. doi: 10.18632/oncotarget.v8i53 PMC5710901 29207620

[13] Chang X Xu B Wang L Wang Y Wang Y Yan S. Investigating a pathogenic role for TXNDC5 in tumors. Int J Oncol. (2013) 43:1871–84. doi: 10.3892/ijo.2013.2123 24100949

[14] Chang X Zhao Y Yan X Pan J Fang K Wang L. Investigating a pathogenic role for TXNDC5 in rheumatoid arthritis. Arthritis Res Ther. (2011) 13:R124. doi: 10.1186/ar3429 21801346 PMC3239364

[15] Jeong KH Shin MK Uhm YK Kim HJ Chung JH Lee MH. Association of TXNDC5 gene polymorphisms and susceptibility to nonsegmental vitiligo in the Korean population. Br J Dermatol. (2010) 162:759–64. doi: 10.1111/j.1365-2133.2009.09574.x 19906073

[16] Lin SH Liu CM Liu YL Shen-Jang Fann C Hsiao PC Wu JY. Clustering by neurocognition for fine mapping of the schizophrenia susceptibility loci on chromosome 6p. Genes Brain Behavior. (2009) 8:785–94. doi: 10.1111/j.1601-183X.2009.00523.x PMC4286260 19694819

[17] Alanen HI Salo KE Pekkala M Siekkinen HM Pirneskoski A Ruddock LW. Defining the domain boundaries of the human protein disulfide isomerases. Antioxid Redox Signal. (2003) 5:367–74. doi: 10.1089/152308603768295096 13678523

[18] Matsusaki M Kanemura S Kinoshita M Lee YH Inaba K Okumura M. The Protein Disulfide Isomerase Family: from proteostasis to pathogenesis. Biochim Biophys Acta Gen Subj. (2020) 1864:129338. doi: 10.1016/j.bbagen.2019.04.003 30986509

[19] Wang C Li W Ren J Fang J Ke H Gong W. Structural insights into the redox-regulated dynamic conformations of human protein disulfide isomerase. Antioxid Redox Signal. (2013) 19:36–45. doi: 10.1089/ars.2012.4630 22657537

[20] Tian G Xiang S Noiva R Lennarz W Schindelin H. The crystal structure of yeast protein disulfide isomerase suggests cooperativity between its active sites. Cell. (2006) 124:61–73. doi: 10.1016/j.cell.2005.10.044 16413482

[21] Klappa P Ruddock LW Darby NJ Freedman RB. The b' domain provides the principal peptide-binding site of protein disulfide isomerase but all domains contribute to binding of misfolded proteins. EMBO J. (1998) 17:927–35. doi: 10.1093/emboj/17.4.927 PMC1170442 9463371

[22] Byrne LJ Sidhu A Wallis AK Ruddock LW Freedman RB Howard MJ. Mapping of the ligand-binding site on the b' domain of human PDI: interaction with peptide ligands and the x-linker region. Biochem J. (2009) 423:209–17. doi: 10.1042/BJ20090565 19604149

[23] Pirneskoski A Klappa P Lobell M Williamson RA Byrne L Alanen HI. Molecular characterization of the principal substrate binding site of the ubiquitous folding catalyst protein disulfide isomerase. J Biol Chem. (2004) 279:10374–81. doi: 10.1074/jbc.M312193200 14684740

[24] Denisov AY Määttänen P Dabrowski C Kozlov G Thomas DY Gehring K. Solution structure of the bb' domains of human protein disulfide isomerase. FEBS J. (2009) 276:1440–9. doi: 10.1111/j.1742-4658.2009.06884.x 19187238

[25] Irvine AG Wallis AK Sanghera N Rowe ML Ruddock LW Howard MJ. Protein disulfide-isomerase interacts with a substrate protein at all stages along its folding pathway. PLoS One. (2014) 9:e82511. doi: 10.1371/journal.pone.0082511 24465374 PMC3896340

[26] Kojima R Okumura M Masui S Kanemura S Inoue M Saiki M. Radically different thioredoxin domain arrangement of ERp46, an efficient disulfide bond introducer of the mammalian PDI family. Structure. (2014) 22:431–43. doi: 10.1016/j.str.2013.12.013 24462249

[27] Sato Y Kojima R Okumura M Hagiwara M Masui S Maegawa K. Synergistic cooperation of PDI family members in peroxiredoxin 4-driven oxidative protein folding. Sci Rep. (2013) 3:2456. doi: 10.1038/srep02456 23949117 PMC3744794

[28] Okumura M Shimamoto S Hidaka Y. A chemical method for investigating disulfide-coupled peptide and protein folding. FEBS J. (2012) 279:2283–95. doi: 10.1111/j.1742-4658.2012.08596.x 22487262

[29] Pace PE Peskin AV Han MH Hampton MB Winterbourn CC. Hyperoxidized peroxiredoxin 2 interacts with the protein disulfide- isomerase ERp46. Biochem J. (2013) 453:475–85. doi: 10.1042/BJ20130030 23713588

[30] Zito E Melo EP Yang Y Wahlander Å Neubert TA Ron D. Oxidative protein folding by an endoplasmic reticulum-localized peroxiredoxin. Mol Cell. (2010) 40:787–97. doi: 10.1016/j.molcel.2010.11.010 PMC3026605 21145486

[31] Tavender TJ Springate JJ Bulleid NJ. Recycling of peroxiredoxin IV provides a novel pathway for disulphide formation in the endoplasmic reticulum. EMBO J. (2010) 29:4185–97. doi: 10.1038/emboj.2010.273 PMC3018787 21057456

[32] Wang X Wang L Wang X Sun F Wang CC. Structural insights into the peroxidase activity and inactivation of human peroxiredoxin 4. Biochem J. (2012) 441:113–8. doi: 10.1042/BJ20110380 21916849

[33] Cao Z Tavender TJ Roszak AW Cogdell RJ Bulleid NJ. Crystal structure of reduced and of oxidized peroxiredoxin IV enzyme reveals a stable oxidized decamer and a non-disulfide-bonded intermediate in the catalytic cycle. J Biol Chem. (2011) 286:42257–66. doi: 10.1074/jbc.M111.298810 PMC3234919 21994946

[34] Rittirsch D Flierl MA Ward PA. Harmful molecular mechanisms in sepsis. Nat Rev Immunol. (2008) 8:776–87. doi: 10.1038/nri2402 PMC2786961 18802444

[35] van der Poll T van de Veerdonk FL Scicluna BP Netea MG. The immunopathology of sepsis and potential therapeutic targets. Nat Rev Immunol. (2017) 17:407–20. doi: 10.1038/nri.2017.36 28436424

[36] Pool R Gomez H Kellum JA. Mechanisms of organ dysfunction in sepsis. Crit Care clinics. (2018) 34:63–80. doi: 10.1016/j.ccc.2017.08.003 PMC6922007 29149942

[37] Lelubre C Vincent JL. Mechanisms and treatment of organ failure in sepsis. Nat Rev Nephrol. (2018) 14:417–27. doi: 10.1038/s41581-018-0005-7 29691495

[38] Chaudhry H Zhou J Zhong Y Ali MM McGuire F Nagarkatti PS. Role of cytokines as a double-edged sword in sepsis. In Vivo (Athens Greece). (2013) 27:669–84. PMC4378830 24292568

[39] Cao C Yu M Chai Y. Pathological alteration and therapeutic implications of sepsis-induced immune cell apoptosis. Cell Death disease. (2019) 10:782. doi: 10.1038/s41419-019-2015-1 31611560 PMC6791888

[40] Wiersinga WJ Leopold SJ Cranendonk DR van der Poll T. Host innate immune responses to sepsis. Virulence. (2013) 5:36–44. doi: 10.4161/viru.25436 23774844 PMC3916381

[41] van der Poll T Opal SM. Host-pathogen interactions in sepsis. Lancet Infect diseases. (2008) 8:32–43. doi: 10.1016/S1473-3099(07)70265-7 18063412

[42] Nissinen L Kähäri VM. Matrix metalloproteinases in inflammation. Biochim Biophys Acta. (2014) 1840:2571–80. doi: 10.1016/j.bbagen.2014.03.007 24631662

[43] Koehne CH Dubois RN. COX-2 inhibition and colorectal cancer. Semin Oncol. (2004) 31:12–21. doi: 10.1053/j.seminoncol.2004.03.041 15252926

[44] Rius J Guma M Schachtrup C Akassoglou K Zinkernagel AS Nizet V. NF-kappaB links innate immunity to the hypoxic response through transcriptional regulation of HIF-1alpha. Nature. (2008) 453:807–11. doi: 10.1038/nature06905 PMC2669289 18432192

[45] Courtois G Gilmore TD. Mutations in the NF-kappaB signaling pathway: implications for human disease. Oncogene. (2006) 25:6831–43. doi: 10.1038/sj.onc.1209939 17072331

[46] Mantovani A Allavena P Sica A Balkwill F. Cancer-related inflammation. Nature. (2008) 454:436–44. doi: 10.1038/nature07205 18650914

[47] Karin M. Nuclear factor-kappaB in cancer development and progression. Nature. (2006) 441:431–6. doi: 10.1038/nature04870 16724054

[48] Zeng Y Ma W Ma C Ren X Wang Y. Inhibition of TXNDC5 attenuates lipopolysaccharide-induced septic shock by altering inflammatory responses. Lab Invest. (2022) 102:422–31. doi: 10.1038/s41374-021-00711-5 34864825

[49] Deane KD O'Donnell CI Hueber W Majka DS Lazar AA Derber LA. The number of elevated cytokines and chemokines in preclinical seropositive rheumatoid arthritis predicts time to diagnosis in an age-dependent manner. Arthritis rheumatism. (2010) 62:3161–72. doi: 10.1002/art.27638 PMC2980824 20597112

[50] Sokolove J Bromberg R Deane KD Lahey LJ Derber LA Chandra PE. Autoantibody epitope spreading in the pre-clinical phase predicts progression to rheumatoid arthritis. PLoS One. (2012) 7:e35296. doi: 10.1371/journal.pone.0035296 22662108 PMC3360701

[51] Kokkonen H Söderström I Rocklöv J Hallmans G Lejon K Rantapää Dahlqvist S. Up-regulation of cytokines and chemokines predates the onset of rheumatoid arthritis. Arthritis rheumatism. (2010) 62:383–91. doi: 10.1002/art.27186 20112361

[52] Chalan P Bijzet J van den Berg A Kluiver J Kroesen BJ Boots AM. Analysis of serum immune markers in seropositive and seronegative rheumatoid arthritis and in high-risk seropositive arthralgia patients. Sci Rep. (2016) 6:26021. doi: 10.1038/srep26021 27189045 PMC4870704

[53] Wang L Song G Zheng Y Wang D Dong H Pan J. miR-573 is a negative regulator in the pathogenesis of rheumatoid arthritis. Cell Mol Immunol. (2016) 13:839–49. doi: 10.1038/cmi.2015.63 PMC5101444 26166764

[54] Xu B Li J Wu C Liu C Yan X Chang X. CXCL10 and TRAIL are upregulated by TXNDC5 in rheumatoid arthritis fibroblast-like synoviocytes. J Rheumatol. (2018) 45:335–40. doi: 10.3899/jrheum.170170 29247155

[55] Barron L Wynn TA. Fibrosis is regulated by Th2 and Th17 responses and by dynamic interactions between fibroblasts and macrophages. Am J Physiol Gastrointestinal liver Physiol. (2011) 300:G723–8. doi: 10.1152/ajpgi.00414.2010 PMC3302189 21292997

[56] Feng W Li W Liu W Wang F Li Y Yan W. IL-17 induces myocardial fibrosis and enhances RANKL/OPG and MMP/TIMP signaling in isoproterenol-induced heart failure. Exp Mol pathology. (2009) 87:212–8. doi: 10.1016/j.yexmp.2009.06.001 19527710

[57] Meng F Wang K Aoyama T Grivennikov SI Paik Y Scholten D. Interleukin-17 signaling in inflammatory, Kupffer cells, and hepatic stellate cells exacerbates liver fibrosis in mice. Gastroenterology. (2012) 143:765–76.e3. doi: 10.1053/j.gastro.2012.05.049 22687286 PMC3635475

[58] Park MJ Moon SJ Lee EJ Jung KA Kim EK Kim DS. IL-1-IL-17 signaling axis contributes to fibrosis and inflammation in two different murine models of systemic sclerosis. Front Immunol. (2018) 9:1611. doi: 10.3389/fimmu.2018.01611 30042768 PMC6048384

[59] Sun B Wang H Zhang L Yang X Zhang M Zhu X. Role of interleukin 17 in TGF-β signaling-mediated renal interstitial fibrosis. Cytokine. (2018) 106:80–8. doi: 10.1016/j.cyto.2017.10.015 29111086

[60] Wang BZ Wang LP Han H Cao FL Li GY Xu JL. Interleukin-17A antagonist attenuates radiation-induced lung injuries in mice. Exp Lung Res. (2014) 40:77–85. doi: 10.3109/01902148.2013.872210 24446677

[61] Wilson MS Madala SK Ramalingam TR Gochuico BR Rosas IO Cheever AW. Bleomycin and IL-1beta-mediated pulmonary fibrosis is IL-17A dependent. J Exp Med. (2010) 207:535–52. doi: 10.1084/jem.20092121 PMC2839145 20176803

[62] Gieseck RL 3rd Ramalingam TR Hart KM Vannella KM Cantu DA Lu WY. Interleukin-13 activates distinct cellular pathways leading to ductular reaction, steatosis, and fibrosis. Immunity. (2016) 45:145–58. doi: 10.1016/j.immuni.2016.06.009 PMC4956513 27421703

[63] Kaviratne M Hesse M Leusink M Cheever AW Davies SJ McKerrow JH. IL-13 activates a mechanism of tissue fibrosis that is completely TGF-beta independent. J Immunol (Baltimore Md 1950). (2004) 173:4020–9. doi: 10.4049/jimmunol.173.6.4020 15356151

[64] Lee CG Homer RJ Zhu Z Lanone S Wang X Koteliansky V. Interleukin-13 induces tissue fibrosis by selectively stimulating and activating transforming growth factor beta(1). J Exp Med. (2001) 194:809–21. doi: 10.1084/jem.194.6.809 PMC2195954 11560996

[65] Shih YC Chen CL Zhang Y Mellor RL Kanter EM Fang Y. Endoplasmic reticulum protein TXNDC5 augments myocardial fibrosis by facilitating extracellular matrix protein folding and redox-sensitive cardiac fibroblast activation. Circ Res. (2018) 122:1052–68. doi: 10.1161/CIRCRESAHA.117.312130 PMC5899016 29535165

[66] Lee TH Yeh CF Lee YT Shih YC Chen YT Hung CT. Fibroblast-enriched endoplasmic reticulum protein TXNDC5 promotes pulmonary fibrosis by augmenting TGFbeta signaling through TGFBR1 stabilization. Nat Commun. (2020) 11:4254. doi: 10.1038/s41467-020-18047-x 32848143 PMC7449970

[67] Chen YT Jhao PY Hung CT Wu YF Lin SJ Chiang WC. Endoplasmic reticulum protein TXNDC5 promotes renal fibrosis by enforcing TGF-beta signaling in kidney fibroblasts. J Clin Invest. (2021) 131:e143645. doi: 10.1172/JCI143645 33465051 PMC7919722

[68] Hung C-T Su T-H Chen Y-T Wu Y-F Chen Y-T Lin S-J. Targeting ER protein TXNDC5 in hepatic stellate cell mitigates liver fibrosis by repressing non-canonical TGFβ signalling. Gut. (2022) 71:1876–91. doi: 10.1136/gutjnl-2021-325065 34933915

[69] Hung CT Tsai YW Wu YS Yeh CF Yang KC. The novel role of ER protein TXNDC5 in the pathogenesis of organ fibrosis: mechanistic insights and therapeutic implications. J BioMed Sci. (2022) 29:63. doi: 10.1186/s12929-022-00850-x 36050716 PMC9438287

[70] Grivennikov SI Greten FR Karin M. Immunity, inflammation, and cancer. Cell. (2010) 140:883–99. doi: 10.1016/j.cell.2010.01.025 PMC2866629 20303878

[71] Coussens LM Werb Z. Inflammation and cancer. Nature. (2002) 420:860–7. doi: 10.1038/nature01322 PMC2803035 12490959

[72] Crusz SM Balkwill FR. Inflammation and cancer: advances and new agents. Nat Rev Clin Oncol. (2015) 12:584–96. doi: 10.1038/nrclinonc.2015.105 26122183

[73] Elinav E Nowarski R Thaiss CA Hu B Jin C Flavell RA. Inflammation-induced cancer: crosstalk between tumours, immune cells and microorganisms. Nat Rev Cancer. (2013) 13:759–71. doi: 10.1038/nrc3611 24154716

[74] Shacter E Weitzman SA. Chronic inflammation and cancer. Oncol (Williston Park NY). (2002) 16:217–26, 29. 11866137

[75] Schaue D Micewicz ED Ratikan JA Xie MW Cheng G McBride WH. Radiation and inflammation. Semin Radiat Oncol. (2015) 25:4–10. doi: 10.1016/j.semradonc.2014.07.007 25481260 PMC4378687

[76] Guthrie GJ Charles KA Roxburgh CS Horgan PG McMillan DC Clarke SJ. The systemic inflammation-based neutrophil-lymphocyte ratio: experience in patients with cancer. Crit Rev oncology/hematology. (2013) 88:218–30. doi: 10.1016/j.critrevonc.2013.03.010 23602134

[77] Lopetuso LR Chowdhry S Pizarro TT. Opposing functions of classic and novel IL-1 family members in gut health and disease. Front Immunol. (2013) 4:181. doi: 10.3389/fimmu.2013.00181 23847622 PMC3705591

[78] Coffelt SB Kersten K Doornebal CW Weiden J Vrijland K Hau CS. IL-17-producing gammadelta T cells and neutrophils conspire to promote breast cancer metastasis. Nature. (2015) 522:345–8. doi: 10.1038/nature14282 PMC4475637 25822788

[79] Hedrick E Mohankumar K Lacey A Safe S. Inhibition of NR4A1 promotes ROS accumulation and IL24-dependent growth arrest in Rhabdomyosarcoma. Mol Cancer Res. (2019) 17:2221–32. doi: 10.1158/1541-7786.MCR-19-0408 PMC6825581 31462501

[80] Gupta P Su ZZ Lebedeva IV Sarkar D Sauane M Emdad L. mda-7/IL-24: multifunctional cancer-specific apoptosis-inducing cytokine. Pharmacol Ther. (2006) 111:596–628. doi: 10.1016/j.pharmthera.2005.11.005 16464504 PMC1781515

[81] Menezes ME Bhoopathi P Pradhan AK Emdad L Das SK Guo C. Role of MDA-7/IL-24 a multifunction protein in human diseases. Adv Cancer Res. (2018) 138:143–82. doi: 10.1016/bs.acr.2018.02.005 PMC6218935 29551126

[82] Bhoopathi P Lee N Pradhan AK Shen XN Das SK Sarkar D. mda-7/IL-24 induces cell death in neuroblastoma through a novel mechanism involving AIF and ATM. Cancer Res. (2016) 76:3572–82. doi: 10.1158/0008-5472.CAN-15-2959 PMC4911293 27197168

[83] Aggarwal BB. Signalling pathways of the TNF superfamily: a double-edged sword. Nat Rev Immunol. (2003) 3:745–56. doi: 10.1038/nri1184 12949498

[84] Sun SC. Non-canonical NF-κB signaling pathway. Cell Res. (2011) 21:71–85. doi: 10.1038/cr.2010.177 21173796 PMC3193406

[85] Veale DJ Fearon U. Inhibition of angiogenic pathways in rheumatoid arthritis: potential for therapeutic targeting. Best Pract Res Clin Rheumatol. (2006) 20:941–7. doi: 10.1016/j.berh.2006.05.004 16980216

[86] Smith MD. The normal synovium. Open Rheumatol J. (2011) 5:100–6. doi: 10.2174/1874312901105010100 PMC3263506 22279508

[87] Hu XX Wu YJ Zhang J Wei W. T-cells interact with B cells, dendritic cells, and fibroblast-like synoviocytes as hub-like key cells in rheumatoid arthritis. Int immunopharmacology. (2019) 70:428–34. doi: 10.1016/j.intimp.2019.03.008 30856393

[88] Muz B Khan MN Kiriakidis S Paleolog EM. Hypoxia. The role of hypoxia and HIF-dependent signalling events in rheumatoid arthritis. Arthritis Res Ther. (2009) 11:201. doi: 10.1186/ar2568 19222864 PMC2688222

[89] Chang X Cui Y Zong M Zhao Y Yan X Chen Y. Identification of proteins with increased expression in rheumatoid arthritis synovial tissues. J Rheumatol. (2009) 36:872–80. doi: 10.3899/jrheum.080939 19369474

[90] Wang L Zheng Y Xu H Yan X Chang X. Investigate pathogenic mechanism of TXNDC5 in rheumatoid arthritis. PloS One. (2013) 8:e53301. doi: 10.1371/journal.pone.0053301 23326410 PMC3541148

[91] Li J Chen Y Liu Q Tian Z Zhang Y. Mechanistic and therapeutic links between rheumatoid arthritis and diabetes mellitus. Clin Exp Med. (2023) 23:287–99. doi: 10.1007/s10238-022-00816-1 35306615

[92] Sampath Kumar A Maiya AG Shastry BA Vaishali K Ravishankar N Hazari A. Exercise and insulin resistance in type 2 diabetes mellitus: A systematic review and meta-analysis. Ann Phys Rehabil Med. (2019) 62:98–103. doi: 10.1016/j.rehab.2018.11.001 30553010

[93] Pi H Zhou H Jin H Ning Y Wang Y. Abnormal glucose metabolism in rheumatoid arthritis. BioMed Res Int. (2017) 2017:9670434. doi: 10.1155/2017/9670434 28529957 PMC5424188

[94] Nicolau J Lequerré T Bacquet H Vittecoq O. Rheumatoid arthritis, insulin resistance, and diabetes. Joint Bone spine. (2017) 84:411–6. doi: 10.1016/j.jbspin.2016.09.001 27777170

[95] Gallagher L Cregan S Biniecka M Cunningham C Veale DJ Kane DJ. Insulin-resistant pathways are associated with disease activity in rheumatoid arthritis and are subject to disease modification through metabolic reprogramming: A potential novel therapeutic approach. Arthritis Rheumatol (Hoboken NJ). (2020) 72:896–902. doi: 10.1002/art.41190 31840936

[96] Ormseth MJ Stein CM. Is visceral fat the missing link in the relationship between inflammation and insulin resistance in RA? J Rheumatol. (2014) 41:1906–9. doi: 10.3899/jrheum.140780 25275092

[97] Quevedo-Abeledo JC Sánchez-Pérez H Tejera-Segura B de Armas-Rillo L Ojeda S Erausquin C. Higher prevalence and degree of insulin resistance in patients with rheumatoid arthritis than in patients with systemic Lupus Erythematosus. J Rheumatol. (2021) 48:339–47. doi: 10.3899/jrheum.200435 32541071

[98] Li AH Morrison AC Kovar C Cupples LA Brody JA Polfus LM. Analysis of loss-of-function variants and 20 risk factor phenotypes in 8,554 individuals identifies loci influencing chronic disease. Nat Genet. (2015) 47:640–2. doi: 10.1038/ng.3270 PMC4470468 25915599

[99] Conrad CH Brooks WW Hayes JA Sen S Robinson KG Bing OH. Myocardial fibrosis and stiffness with hypertrophy and heart failure in the spontaneously hypertensive rat. Circulation. (1995) 91:161–70. doi: 10.1161/01.CIR.91.1.161 7805198

[100] Schwarz F Mall G Zebe H Blickle J Derks H Manthey J. Quantitative morphologic findings of the myocardium in idiopathic dilated cardiomyopathy. Am J Cardiol. (1983) 51:501–6. doi: 10.1016/S0002-9149(83)80088-5 6218745

[101] Greenberg B Quinones MA Koilpillai C Limacher M Shindler D Benedict C. Effects of long-term enalapril therapy on cardiac structure and function in patients with left ventricular dysfunction. Results of the SOLVD echocardiography substudy. Circulation. (1995) 91:2573–81. doi: 10.1161/01.CIR.91.10.2573 7743619

[102] Zannad F Alla F Dousset B Perez A Pitt B. Limitation of excessive extracellular matrix turnover may contribute to survival benefit of spironolactone therapy in patients with congestive heart failure: insights from the randomized aldactone evaluation study (RALES). Rales Investigators Circulation. (2000) 102:2700–6. doi: 10.1161/01.CIR.102.22.2700 11094035

[103] Tsutamoto T Wada A Maeda K Mabuchi N Hayashi M Tsutsui T. Effect of spironolactone on plasma brain natriuretic peptide and left ventricular remodeling in patients with congestive heart failure. J Am Coll Cardiol. (2001) 37:1228–33. doi: 10.1016/S0735-1097(01)01116-0 11300427

[104] Lee KW Everett T Rahmutula D Guerra JM Wilson E Ding C. Pirfenidone prevents the development of a vulnerable substrate for atrial fibrillation in a canine model of heart failure. Circulation. (2006) 114:1703–12. doi: 10.1161/CIRCULATIONAHA.106.624320 PMC2129103 17030685

[105] Martin J Kelly DJ Mifsud SA Zhang Y Cox AJ See F. Tranilast attenuates cardiac matrix deposition in experimental diabetes: role of transforming growth factor-beta. Cardiovasc Res. (2005) 65:694–701. doi: 10.1016/j.cardiores.2004.10.041 15664396

[106] Holmes JS Arispe IE Moy E. Heart disease and prevention: race and age differences in heart disease prevention, treatment, and mortality. Med Care. (2005) 43:I33–41. doi: 10.1097/00005650-200503001-00006 15746589

[107] Derynck R Zhang YE. Smad-dependent and Smad-independent pathways in TGF-beta family signalling. Nature. (2003) 425:577–84. doi: 10.1038/nature02006 14534577

[108] Leask A Abraham DJ. TGF-beta signaling and the fibrotic response. FASEB J. (2004) 18:816–27. doi: 10.1096/fj.03-1273rev 15117886

[109] Mora AL Rojas M Pardo A Selman M. Emerging therapies for idiopathic pulmonary fibrosis, a progressive age-related disease. Nat Rev Drug discovery. (2017) 16:810. doi: 10.1038/nrd.2017.225 PMC5815310 29081515

[110] Herbertz S Sawyer JS Stauber AJ Gueorguieva I Driscoll KE Estrem ST. Clinical development of galunisertib (LY2157299 monohydrate), a small molecule inhibitor of transforming growth factor-beta signaling pathway. Drug design Dev Ther. (2015) 9:4479–99. doi: 10.2147/DDDT PMC4539082 26309397

[111] Anderton MJ Mellor HR Bell A Sadler C Pass M Powell S. Induction of heart valve lesions by small-molecule ALK5 inhibitors. Toxicologic pathology. (2011) 39:916–24. doi: 10.1177/0192623311416259 21859884

[112] Shull MM Ormsby I Kier AB Pawlowski S Diebold RJ Yin M. Targeted disruption of the mouse transforming growth factor-beta 1 gene results in multifocal inflammatory disease. Nature. (1992) 359:693–9. doi: 10.1038/359693a0 PMC3889166 1436033

[113] Kulkarni AB Huh CG Becker D Geiser A Lyght M Flanders KC. Transforming growth factor beta 1 null mutation in mice causes excessive inflammatory response and early death. Proc Natl Acad Sci U S A. (1993) 90:770–4. doi: 10.1073/pnas.90.2.770 PMC45747 8421714

[114] Xing Y Li C Li A Sridurongrit S Tiozzo C Bellusci S. Signaling *via* Alk5 controls the ontogeny of lung Clara cells. Dev (Cambridge England). (2010) 137:825–33. doi: 10.1242/dev.040535 PMC2827691 20147383

[115] Saito A Horie M Nagase T. TGF-β Signaling in lung health and disease. Int J Mol Sci. (2018) 19:2460. doi: 10.3390/ijms19082460 30127261 PMC6121238

[116] Li L Fu H Liu Y. The fibrogenic niche in kidney fibrosis: components and mechanisms. Nat Rev Nephrol. (2022) 18:545–57. doi: 10.1038/s41581-022-00590-z 35788561

[117] Meng XM Nikolic-Paterson DJ Lan HY. TGF-beta: the master regulator of fibrosis. Nat Rev Nephrol. (2016) 12:325–38. doi: 10.1038/nrneph.2016.48 27108839

[118] Liu BC Tang TT Lv LL Lan HY. Renal tubule injury: a driving force toward chronic kidney disease. Kidney Int. (2018) 93:568–79. doi: 10.1016/j.kint.2017.09.033 29361307

[119] Macconi D Remuzzi G Benigni A. Key fibrogenic mediators: old players. Renin-angiotensin system. Kidney Int supplements. (2014) 4:58–64. doi: 10.1038/kisup.2014.11 PMC4536968 26312151

[120] Yang L Besschetnova TY Brooks CR Shah JV Bonventre JV. Epithelial cell cycle arrest in G2/M mediates kidney fibrosis after injury. Nat Med. (2010) 16:535–43, 1p following 143. doi: 10.1038/nm.2144 20436483 PMC3928013

[121] Kendall RT Feghali-Bostwick CA. Fibroblasts in fibrosis: novel roles and mediators. Front Pharmacol. (2014) 5:123. doi: 10.3389/fphar.2014.00123 24904424 PMC4034148

[122] Marcellin P Kutala BK. Liver diseases: A major, neglected global public health problem requiring urgent actions and large-scale screening. Liver Int. (2018) 38 Suppl 1:2–6. doi: 10.1111/liv.13682 29427496

[123] Friedman SL. Liver fibrosis – from bench to bedside. J hepatology. (2003) 38 Suppl 1:S38–53. doi: 10.1016/S0168-8278(02)00429-4 12591185

[124] Bataller R Brenner DA. Liver fibrosis. J Clin Invest. (2005) 115:209–18. doi: 10.1172/JCI24282 PMC546435 15690074

[125] Ginès P Cárdenas A Arroyo V Rodés J. Management of cirrhosis and ascites. New Engl J Med. (2004) 350:1646–54. doi: 10.1056/NEJMra035021 15084697

[126] Tsuchida T Friedman SL. Mechanisms of hepatic stellate cell activation. Nat Rev Gastroenterol hepatology. (2017) 14:397–411. doi: 10.1038/nrgastro.2017.38 28487545

[127] Robles J Prakash A Vizcaíno JA Casal JI. Integrated meta-analysis of colorectal cancer public proteomic datasets for biomarker discovery and validation. PLoS Comput Biol. (2024) 20:e1011828. doi: 10.1371/journal.pcbi.1011828 38252632 PMC10833860

[128] Chawsheen HA Ying Q Jiang H Wei Q. A critical role of the thioredoxin domain containing protein 5 (TXNDC5) in redox homeostasis and cancer development. Genes Dis. (2018) 5:312–22. doi: 10.1016/j.gendis.2018.09.003 PMC6303481 30591932

[129] Wang L Song G Chang X Tan W Pan J Zhu X. The role of TXNDC5 in castration-resistant prostate cancer-involvement of androgen receptor signaling pathway. Oncogene. (2015) 34:4735–45. doi: 10.1038/onc.2014.401 25500540

[130] Tan F Zhu H He X Yu N Zhang X Xu H. Role of TXNDC5 in tumorigenesis of colorectal cancer cells: *In vivo* and in *vitro* evidence. Int J Mol Med. (2018) 42:935–45. doi: 10.3892/ijmm PMC6034924 29749460

[131] Chawsheen HA Jiang H Ying Q Ding N Thapa P Wei Q. The redox regulator sulfiredoxin forms a complex with thioredoxin domain-containing 5 protein in response to ER stress in lung cancer cells. J Biol Chem. (2019) 294:8991–9006. doi: 10.1074/jbc.RA118.005804 31000628 PMC6552416

[132] Batool S Bin-T-Abid D Batool H Shahid S Saleem M Khan A. Development of multi-epitope vaccine constructs for non-small cell lung cancer (NSCLC) against USA human leukocyte antigen background: an immunoinformatic approach toward future vaccine designing. Expert Opin Biol Ther. (2021) 21:1525–33. doi: 10.1080/14712598.2021.1981285 34547976

[133] Wu Z Zhang L Li N Sha L Zhang K. An immunohistochemical study of thioredoxin domain-containing 5 expression in gastric adenocarcinoma. Oncol Lett. (2015) 9:1154–8. doi: 10.3892/ol.2014.2832 PMC4315038 25663872

[134] Zhang L Hou Y Li N Wu K Zhai J. The influence of TXNDC5 gene on gastric cancer cell. J Cancer Res Clin Oncol. (2010) 136:1497–505. doi: 10.1007/s00432-010-0807-x PMC11827854 20157732

[135] Wang H Yang X Guo Y Shui L Li S Bai Y. HERG1 promotes esophageal squamous cell carcinoma growth and metastasis through TXNDC5 by activating the PI3K/AKT pathway. J Exp Clin Cancer Res. (2019) 38:324. doi: 10.1186/s13046-019-1284-y 31331361 PMC6647263

[136] Zang H Li Y Zhang X Huang G. Circ_0000517 Contributes to Hepatocellular Carcinoma Progression by Upregulating TXNDC5 *via* Sponging miR-1296-5p. Cancer Manag Res. (2020) 12:3457–68. doi: 10.2147/CMAR.S244024 PMC7234960 32523376

[137] Vincent EE Elder DJ Phillips L Heesom KJ Pawade J Luckett M. Overexpression of the TXNDC5 protein in non-small cell lung carcinoma. Anticancer Res. (2011) 31:1577–82. 21617212

[138] Kocatürk B. Identification of thioredoxin domain containing family members' expression pattern and prognostic value in diffuse gliomas *via* in silico analysis. Cancer Med. (2023) 12:3830–44. doi: 10.1002/cam4.5169 PMC9939227 36106447

[139] Malhotra JD Kaufman RJ. Endoplasmic reticulum stress and oxidative stress: a vicious cycle or a double-edged sword? Antioxid Redox Signal. (2007) 9:2277–93. doi: 10.1089/ars.2007.1782 17979528

[140] Du Q Huo F Du W Sun X Jiang X Zhang L. METTL3 potentiates progression of cervical cancer by suppressing ER stress *via* regulating m6A modification of TXNDC5 mRNA. Oncogene. (2022) 41:4420–32. doi: 10.1038/s41388-022-02435-2 35987795

[141] Yu J Yang M Zhou B Luo J Zhang Z Zhang W. CircRNA-104718 acts as competing endogenous RNA and promotes hepatocellular carcinoma progression through microRNA-218-5p/TXNDC5 signaling pathway. Clin Sci (Lond). (2019) 133:1487–503. doi: 10.1042/CS20190394 31278132

[142] Ge Y Jia B Zhang P Chen B Liu L Shi Y. TBX15 facilitates Malignant progression of glioma by transcriptional activation of TXDNC5. iScience. (2024) 27:108950. doi: 10.1016/j.isci.2024.108950 38327797 PMC10847739

[143] Mo R Peng J Xiao J Ma J Li W Wang J. High TXNDC5 expression predicts poor prognosis in renal cell carcinoma. Tumor Biol. (2016) 37:9797–806. doi: 10.1007/s13277-016-4891-7 26810069

[144] Chen X Cubillos-Ruiz JR. Endoplasmic reticulum stress signals in the tumour and its microenvironment. Nat Rev Cancer. (2021) 21:71–88. doi: 10.1038/s41568-020-00312-2 33214692 PMC7927882

[145] Lee SO Jin UH Kang JH Kim SB Guthrie AS Sreevalsan S. The orphan nuclear receptor NR4A1 (Nur77) regulates oxidative and endoplasmic reticulum stress in pancreatic cancer cells. Mol Cancer Res. (2014) 12:527–38. doi: 10.1158/1541-7786.MCR-13-0567 PMC4407472 24515801

[146] Duivenvoorden WC Paschos A Hopmans SN Austin RC Pinthus JH. Endoplasmic reticulum protein ERp46 in renal cell carcinoma. PLoS One. (2014) 9:e90389. doi: 10.1371/journal.pone.0090389 24594673 PMC3940878

[147] Wei Q Jiang H Xiao Z Baker A Young MR Veenstra TD. Sulfiredoxin–Peroxiredoxin IV axis promotes human lung cancer progression through modulation of specific phosphokinase signaling. Proc Natl Acad Sci. (2011) 108:7004–9. doi: 10.1073/pnas.1013012108 PMC3084097 21487000

[148] Yeh CF Cheng SH Lin YS Shentu TP Huang RT Zhu J. Targeting mechanosensitive endothelial TXNDC5 to stabilize eNOS and reduce atherosclerosis in vivo. Sci Adv. (2022) 8:eabl8096. doi: 10.1126/sciadv.abl8096 35061532 PMC8782452

[149] Kuhlencordt PJ Gyurko R Han F Scherrer-Crosbie M Aretz TH Hajjar R. Accelerated atherosclerosis, aortic aneurysm formation, and ischemic heart disease in apolipoprotein E/endothelial nitric oxide synthase double-knockout mice. Circulation. (2001) 104:448–54. doi: 10.1161/hc2901.091399 11468208

[150] Holmgren A. Thioredoxin catalyzes the reduction of insulin disulfides by dithiothreitol and dihydrolipoamide. J Biol Chem. (1979) 254:9627–32. doi: 10.1016/S0021-9258(19)83562-7 385588

[151] Ramirez-Torres A Barcelo-Batllori S Martinez-Beamonte R Navarro MA Surra JC Arnal C. Proteomics and gene expression analyses of squalene-supplemented mice identify microsomal thioredoxin domain-containing protein 5 changes associated with hepatic steatosis. J Proteomics. (2012) 77:27–39. doi: 10.1016/j.jprot.2012.07.001 22796066

[152] Karatas E Raymond AA Leon C Dupuy JW Di-Tommaso S Senant N. Hepatocyte proteomes reveal the role of protein disulfide isomerase 4 in alpha 1-antitrypsin deficiency. JHEP Rep. (2021) 3:100297. doi: 10.1016/j.jhepr.2021.100297 34151245 PMC8192868

[153] Chen L Borozan I Sun J Guindi M Fischer S Feld J. Cell-type specific gene expression signature in liver underlies response to interferon therapy in chronic hepatitis C infection. Gastroenterology. (2010) 138:1123–33.e1-3. doi: 10.1053/j.gastro.2009.10.046 19900446

[154] Chen L Sun J Meng L Heathcote J Edwards AM McGilvray ID. ISG15, a ubiquitin-like interferon-stimulated gene, promotes hepatitis C virus production in *vitro*: implications for chronic infection and response to treatment. J Gen virol. (2010) 91:382–8. doi: 10.1099/vir.0.015388-0 19846672

[155] Real CI Megger DA Sitek B Jahn-Hofmann K Ickenstein LM John MJ. Identification of proteins that mediate the pro-viral functions of the interferon stimulated gene 15 in hepatitis C virus replication. Antiviral Res. (2013) 100:654–61. doi: 10.1016/j.antiviral.2013.10.009 24416772

[156] Kim SH Kim JH Suk JM Lee YI Kim J Lee JH. Identification of skin aging biomarkers correlated with the biomechanical properties. Skin Res Technol. (2021) 27:940–7. doi: 10.1111/srt.13046 33891336

[157] Noh JY Oh SH Lee JH Kwon YS Ryu DJ Lee KH. Can blood components with age-related changes influence the ageing of endothelial cells? Exp Dermatol. (2010) 19:339–46. doi: 10.1111/j.1600-0625.2009.01010.x 19925638

[158] Bae H Lunetta KL Murabito JM Andersen SL Schupf N Perls T. Genetic associations with age of menopause in familial longevity. Menopause. (2019) 26:1204–12. doi: 10.1097/GME.0000000000001367 PMC7008937 31188284

[159] Andrews JM Schmidt JA Carson KR Musiek AC Mehta-Shah N Payton JE. Novel cell adhesion/migration pathways are predictive markers of HDAC inhibitor resistance in cutaneous T cell lymphoma. EBioMedicine. (2019) 46:170–83. doi: 10.1016/j.ebiom.2019.07.053 PMC6711861 31358475

[160] Dytfeld D Luczak M Wrobel T Usnarska-Zubkiewicz L Brzezniakiewicz K Jamroziak K. Comparative proteomic profiling of refractory/relapsed multiple myeloma reveals biomarkers involved in resistance to bortezomib-based therapy. Oncotarget. (2016) 7:56726–36. doi: 10.18632/oncotarget.v7i35 PMC5302948 27527861

[161] Jung EJ Chung KH Kim CW. Identification of simvastatin-regulated targets associated with JNK activation in DU145 human prostate cancer cell death signaling. BMB Rep. (2017) 50:466–71. doi: 10.5483/BMBRep.2017.50.9.087 PMC5625694 28803608

[162] Gu MX Fu Y Sun XL Ding YZ Li CH Pang W. Proteomic analysis of endothelial lipid rafts reveals a novel role of statins in antioxidation. J Proteome Res. (2012) 11:2365–73. doi: 10.1021/pr300098f 22428589

[163] Huang D Liu AYN Leung KS Tang NLS. Direct measurement of B lymphocyte gene expression biomarkers in peripheral blood transcriptomics enables early prediction of vaccine seroconversion. Genes. (2021) 12:971. doi: 10.3390/genes12070971 34202032 PMC8304400

